# Total Structure, Structural Transformation and Catalytic Hydrogenation of [Cu_41_(SC_6_H_3_F_2_)_15_Cl_3_(P(PhF)_3_)_6_(H)_25_]^2−^ Constructed from Twisted Cu_13_ Units

**DOI:** 10.1002/advs.202307085

**Published:** 2023-12-08

**Authors:** Huimin Zhou, Tengfei Duan, Zidong Lin, Tao Yang, Huijuan Deng, Shan Jin, Yong Pei, Manzhou Zhu

**Affiliations:** ^1^ Institutes of Physical Science and Information Technology and Centre for Atomic Engineering of Advanced Materials Key Laboratory of Structure and Functional Regulation of Hybrid Materials of Ministry of Education Department of Chemistry and Anhui Province Key Laboratory of Chemistry for Inorganic/Organic Hybrid Functionalized Materials Anhui University Hefei Anhui 230601 China; ^2^ Department of Chemistry Key Laboratory of Environmentally Friendly Chemistry and Applications of MOE Xiangtan University Xiangtan Hunan 411105 China

**Keywords:** copper nanocluster, electronic structure, high hydrogenation activity, the nanocluster‐to‐nanocluster transformation, twisted Cu_13_ units assemble

## Abstract

Herein, a remarkable achievement in the synthesis and characterization of an atomically precise copper‐hydride nanocluster, [Cu_41_(SC_6_H_3_F_2_)_15_Cl_3_(P(PhF)_3_)_6_(H)_25_]^2−^ via a mild one‐pot reaction is presented. Through X‐ray crystallography analysis, it is revealed that [Cu_41_(SC_6_H_3_F_2_)_15_Cl_3_(P(PhF)_3_)_6_(H)_25_]^2−^ exhibits a unique shell–core–shell structure. The inner Cu_29_ kernel is composed of three twisted Cu_13_ units, connected through Cu_4_ face sharing. Surrounding the metal core, two Cu_6_ metal shells, resembling a protective sandwich structure are observed. This arrangement, along with intracluster *π*···*π* interactions and intercluster C─H···F─C interactions, contributes to the enhanced stability of [Cu_41_(SC_6_H_3_F_2_)_15_Cl_3_(P(PhF)_3_)_6_(H)_25_]^2−^. The presence, number, and location of hydrides within the nanocluster are established through a combination of experimental and density functional theory investigations. Notably, the addition of a phosphine ligand triggers a fascinating nanocluster‐to‐nanocluster transformation in [Cu_41_(SC_6_H_3_F_2_)_15_Cl_3_(P(PhF)_3_)_6_(H)_25_]^2−^, resulting in the generation of two nanoclusters, [Cu_14_(SC_6_H_3_F_2_)_3_(PPh_3_)_8_H_10_]^+^ and [Cu_13_(SC_6_H_3_F_2_)_3_(P(PhF)_3_)_7_H_10_]^0^. Furthermore, it is demonstrated that [Cu_41_(SC_6_H_3_F_2_)_15_Cl_3_(P(PhF)_3_)_6_(H)_25_]^2−^ exhibits catalytic activity in the hydrogenation of nitroarenes. This intriguing nanocluster provides a unique opportunity to explore the assembly of M_13_ units, similar to other coinage metal nanoclusters, and investigate the nanocluster‐to‐nanocluster transformation in phosphine and thiol ligand co‐protected copper nanoclusters.

## Graphical abstract

A novetly copper‐hydride [Cu_41_(SC_6_H_3_F_2_)_15_Cl_3_(P(PhF)_3_)_6_(H)_25_]^2−^cluster is synthesized via a mild method. Via structural analysis, the twisted Cu_13_ unit is capable of constructing a copper core in a coplanar fashion. Various intracluster *π*···*π* and intercluster C─H···F─C interactions contribute to the stability of [Cu_41_(SC_6_H_3_F_2_)_15_Cl_3_(P(PhF)_3_)_6_(H)_25_]^2−^. The introduction of external phosphine ligands can induce a structural transformation of [Cu_41_(SC_6_H_3_F_2_)_15_Cl_3_(P(PhF)_3_)_6_(H)_25_]^2−^. This stable [Cu_41_(SC_6_H_3_F_2_)_15_Cl_3_(P(PhF)_3_)_6_(H)_25_]^2−^ cluster has demonstrated notable hydrogenation activity, which benefit the application in the future.

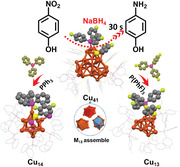

## 1 Introduction

Atomically precise copper hydride nanoclusters have emerged as promising nanomaterials for hydrogen storage and catalysis, similar to gold and silver nanoclusters.^[^ ^1^, ^2^, ^3^, ^4^, ^5^ ^]^ However, unlike the rapid development of gold and silver clusters, the easy oxidation of copper poses a challenge in the synthesis of stable copper clusters. Consequently, the synthesis and characterization of stable copper‐hydride nanoclusters remain a significant challenge. Until now, only a handful of copper‐hydride nanoclusters have been reported, including Cu_11_H_3_(Tf‐dpf)_6_(OAc)_2_/[Cu_12_H_3_(Tf‐dpf)_6_(OAc)_2_]^+^,^[^ ^6^ ^]^ [Cu_15_H_2_(S_2_CNR_2_)_6_(C_2_Ph)_6_]^+^,^[^ ^7^ ^]^ Cu_18_H(PET)_14_(Ph_3_P)_6_(NCS)_6_,^[^ ^8^ ^]^ [Cu_23_(PhSe)_16_(Ph_3_P)_8_H_6_]^+^,^[^ ^9^ ^]^ [Cu_25_H_22_(Ph_3_P)_12_]^+^,^[^ ^10^ ^]^ [Cu_28_H_8_(C_6_H_11_S)_18_(Ph_2_Py)_3_]^2+^/Cu_28_H_10_(C_7_H_7_S)_18_(Ph_3_P)_3_/[Cu_28_H_15_(S_2_CNR)_12_]^+^/[Cu_28_H_20_(S_2_P(OiPr)_2_)_9_]^‐^,^[^ ^11^ ^]^ [Cu_29_H_10_(C_6_H_11_S)_18_(P(Ph‐^p^Me)_3_)_4_]^+^,^[^ ^11a^ ^]^ Cu_30_H_18_(E_2_P(OR)_2_)_12_,^[^ ^12^ ^]^ Cu_31_H_6_(RS)_25_(NHC)_3_,^[^ ^13^ ^]^ [Cu_32_(PET)_24_H_8_Cl_2_]^2‐^,^[^ ^14^ ^]^ Cu_36_H_10_(PET)_24_(Ph_3_P)Cl_2_,^[^ ^15^ ^]^ [Cu_53_(RCOO)_10_(C≡C^t^Bu)_20_Cl_2_H_18_]^+^,^[^ ^16^ ^]^ [Cu_57_H_20_(PET)_36_(Ph_3_P)_4_]^+^,^[^ ^17^ ^]^ [Cu_58_H_20_(PET)_36_(Ph_3_P)_4_]^2+^/[Cu_58_H_20_(SPr)_36_(Ph_3_P)_8_]^2+^,^[^ ^17^, ^18^ ^]^ [Cu_61_(S^t^Bu)_26_S_6_Cl_6_H_14_]^+^^[^ ^19^ ^]^ as well as [Cu_81_(PhS)_46_(^t^BuNH_2_)_10_H_32_]^3+^.^[^ ^20^ ^]^ These copper‐hydride nanoclusters exhibit various core–shell structures, such as the unprecedented planar Cu_17_ core surrounded by a hemispherical shell in Cu_81_,^[^ ^20^ ^]^ the quasi‐J_36_ Cu_19_ core in Cu_61_,^[^ ^19^ ^]^ and Cu_13_ cores capped with different metal shells in others.^[^ ^10^, ^11^ ^]^ The structural information on the core–shell architecture of copper nanoclusters enhances our fundamental understanding of structure‐dependent properties and paves the way for the development of applications in copper nanomaterials.

In the field of cluster development, the evolution of the metal core through internal assembly has been observed, and changes in the assembly of the metal core can significantly impact the overall performance of the cluster.^[^ ^21^, ^22^, ^23^, ^24^, ^25^, ^26^ ^]^ Currently, the presence of M_13_ units has been observed in coinage metal clusters, particularly in gold and silver nanoclusters. These M_13_ units serve as building blocks for constructing the core assembly of the clusters through various mechanisms, such as point sharing, M_3_ plane sharing, and fusion of M_7_ units.^[^ ^22^, ^27^, ^28^, ^29^, ^30^, ^31^, ^32^, ^33^ ^]^ However, such phenomena have not been observed in copper clusters. Therefore, the question arises: can we observe self‐assembly of the inner core in copper clusters based on Cu_13_ units, and if so, how does it compare to gold and silver clusters?

Stable ligands with appropriate electronic effects play a crucial role in guiding the formation of metal nanoclusters. In view of this, various ligands are used to stabilize the structure during the synthesis of clusters.^[^ ^34^, ^35^, ^36^, ^37^, ^38^, ^39^, ^40^ ^]^ For our study, the choice of tris(4‐fluorophenyl)phosphine (P(PhF)_3_) and 2,4‐difluorobenzenethiol (HSC_6_H_3_F_2_) for the synthesis was deliberate and strategic (Details are given in the Experiment Section, Supporting Information). Through this method, we obtained a novel copper‐hydride nanocluster with the chemical formula [Cu_41_(SC_6_H_3_F_2_)_15_Cl_3_(P(PhF)_3_)_6_(H)_25_]^2−^. The crystal structure of the nanocluster was resolved using X‐ray crystallography. The results showed that the nanocluster has a Cu_29_ core assembled by three twisted Cu_13_ units through Cu_4_ face sharing. This core structure is observed for the first time in coinage metal nanoclusters. The Cu_29_ core was further stabilized by two Cu_6_(SR)_6_(PR_3_), three thiol ligands, and three Cl ligands. Moreover, we found that the addition of other PPh_3_ and P(PhF)_3_ ligands can trigger the nanocluster‐to‐nanocluster transformation. This finding provides a new synthesis strategy for regulating the construction of co‐protected copper nanoclusters using phosphine and thiol ligands. Overall, our study highlights the importance of ligands in stabilizing the structure of metal nanoclusters and provides a novel synthesis strategy for regulating the construction of co‐protected copper nanoclusters.

## 2 Results and Discussion

### 2.1 Synthesis, Crystal Structure, and Characterization

**Scheme 1:**
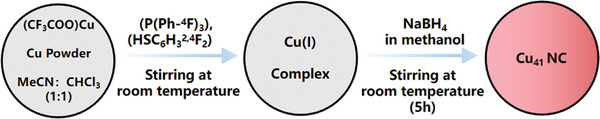
Synthesis of [Cu_41_(SC_6_H_3_F_2_)_15_Cl_3_(P(PhF)_3_)_6_(H)_25_]^2−^ nanocluster.

The synthesis of [Cu_41_(SC_6_H_3_F_2_)_15_Cl_3_(P(PhF)_3_)_6_(H)_25_]^2−^ was successfully achieved under mild conditions using a one‐pot method (**Scheme** **1**). Typically, copper (II) tifluoroacetate hydrate ((CF_3_COO)_2_Cu) and copper powder (Cu) were dissolved in a solvent mixture of acetonitrile and chloroform. Then, tris(4‐fluorophenyl)phosphine (P(PhF)_3_) and 2,4‐difluorobenzenethiol (HSC_6_H_3_F_2_) was added. After 20 mins, a freshly prepared solution of NaBH_4_ was added dropwise, resulting in an immediate formation of a red solution. The reaction was allowed to proceed for 5 h at room temperature. The crude product was obtained by rotary evaporation, followed by dissolution in dichloromethane and acetonitrile, respectively. The supernatant was collected through centrifugation for purification. After 1 week, the crystals were obtained by crystallization from CH_2_Cl_2_/hexane at room temperature, yielding dark red crystals.

**Figure 1:**
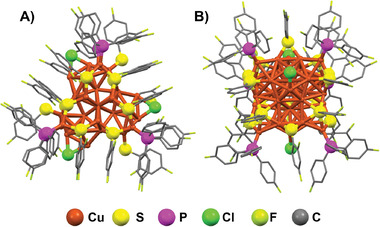
A) Top and B) Side views of the overall structure of Cu_41_. Hydrogen atoms of ligands are omitted for clarity.

The single‐crystal X‐ray crystallographic analysis revealed that the copper nanocluster consisted of 41 Cu atoms, 15 ‐SC_6_H_3_F_2_, 3 Cl, and 6 P(PhF)_3_ ligands (**Figure** **1**). It crystallizes in a monoclinic lattice with a space group of P2_1_/c with lattice parameters a = 20.3129(15), b = 33.065(2), and c = 36.168(2) Å (Table S1, Supporting Information). Interestingly, both enantiomers crystallize as a racemic mixture in the cell units, with a ratio of 1:1 (Figure S1, Supporting Information). The packing mode shows that the left and right structures are closely combined, providing enhanced stability to the nanocluster (Figure S2, Supporting Information). Since determining the presence of hydride in copper‐hydride is highly challenging, we performed electrospray ionization mass spectrometry (ESI–MS) and density function theory (DFT) calculations to further investigate the structure. The overall structure of Cu_41_ exhibits a C_3_ symmetry and a roughly barrel‐like geometry.

**Figure 2:**
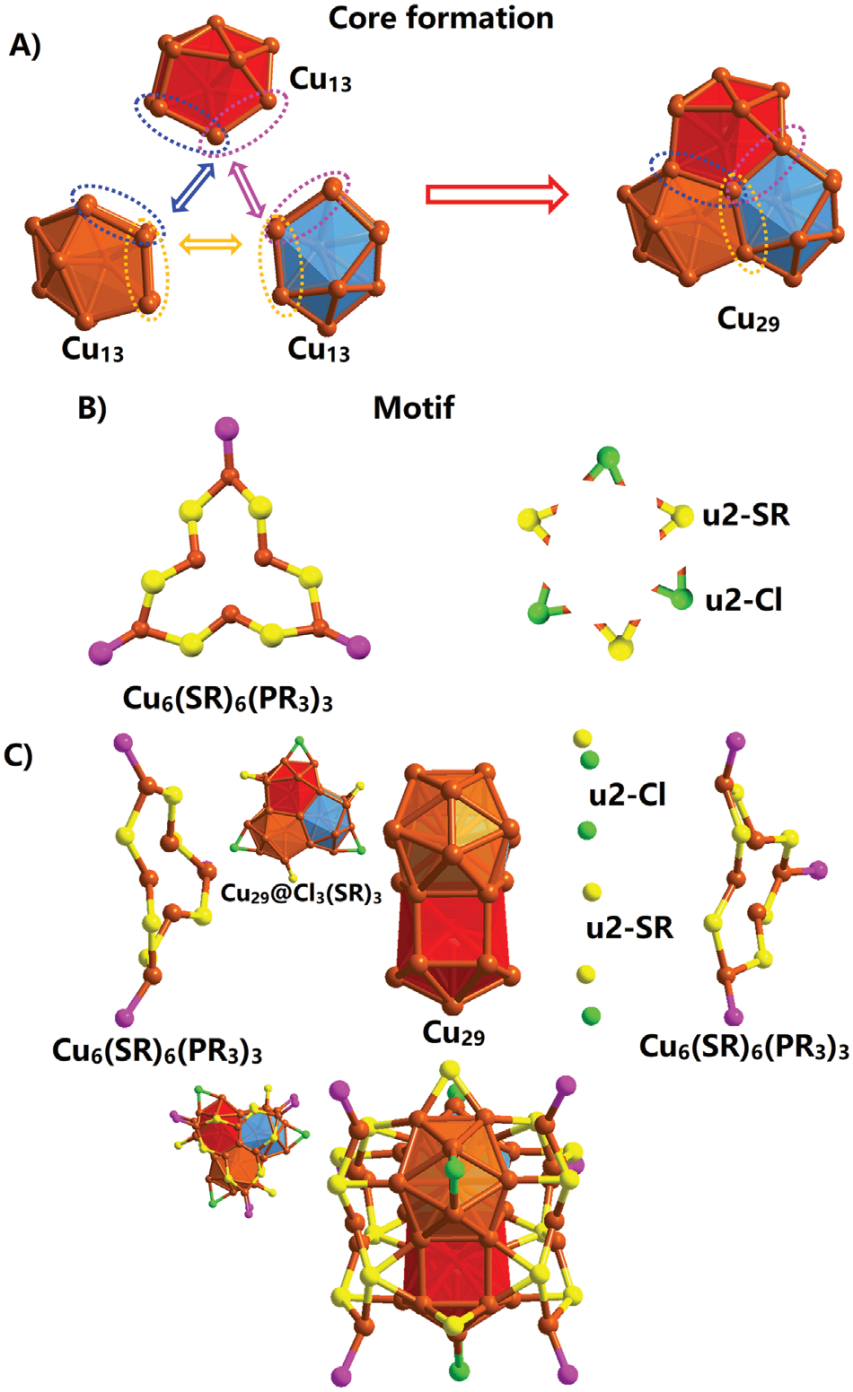
Analysis of the crystal structure of Cu_41_(SR)_15_Cl_3_(PR_3_)_6._ A) Formation of the Cu_29_ core by Cu_4_ sharing of three Cu_13_ units. B) ligand motif of the cluster. C) Linking of motifs with the Cu_29_ core. Carbon and fluorine atoms of ligands are omitted for clarity.

A detailed structure analysis of the cluster revealed that this nanocluster possesses a core–shell structure, consisting of a Cu_29_ core and a Cu_12_(SR)_15_Cl_3_(PR_3_)_6_ shell (**Figure** **2**). Notably, the Cu_29_ core is formed by the self‐assembly of three twisted Cu_13_ units, which come together to create a ring‐shaped structure through Cu_4_ face sharing (Figure 2A). This unique assembly of the core is the first observation of its kind among copper metal nanoclusters, as the common configurations are typically icosahedral or cuboctahedral Cu_13_ cores.^[^ ^41^, ^42^, ^43^ ^]^ The shell of the nanocluster is composed of two complex Cu_6_(SR)_6_(PR_3_)_3_ motifs, along with three thiol ligands and three Cl ligands (Figure 2B). The three thiol ligands and three Cl were present in bridging (µ−2) mode to cap the Cu_29_ core, forming the Cu_29_@Cl_3_(SR)_3_ framework. Finally, the two Cu_6_(SR)_6_(PR_3_)_3_ motifs bind to the Cu_29_@Cl_3_(SR)_3_ structure from its left and right side, completing the overall structure of Cu_41_ (Figure 2C). Consequently, the entire structure can be divided into three layers. As shown in Figure S3 (Supporting Information), the average bond length of the Cu_kernel_–Cu_kernel_ in the assembled Cu_29_ kernel structure was 2.678 Å, with the three twisted Cu_13_ units having slightly different average bond lengths. The average bond length of Cu_kernel_–Cl was 2.346 Å and that of Cu_kernel_–S was 2.320 Å. In the two shell Cu_6_(SR)_6_(PR_3_)_3_ motifs, the average bond length of Cu_shell_–Cu_kernel_ and Cu_kernel_–S_shell_ was 2.680 and 2.301 Å, respectively. The average bond length of Cu_shell_–P was 2.197 Å. The analysis of the bond length data of Cu_41_ clusters can enrich the research of the M_13_ unit planar linear assembly kernel. Linear growth via Au_3_ face‐sharing was observed in Au_38_(SC_2_H_4_Ph)_24_ and Ag_32_, which has a biicosahedral M_23_ kernel. Face‐fused biicosahedral (Pd_23_) and triicosahedral (Pd_33_) kernels were also previously found in Pd_39_(CO)_23_(PMe_3_)_16_ and Pd_69_(CO)_36_(PEt_3_)_18_, respectively.^[^ ^44^, ^45^, ^46^ ^]^ However, reported for the first time in this work is a Cu_4_ tetrahedron‐based implementation of ring growth via face‐sharing.

**Figure 3:**
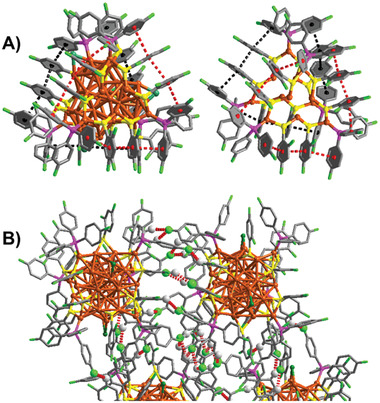
A) The intracluster *π*···*π* interactions in Cu_41_. B) Intercluster C─H···F interactions in the unit cell of Cu_41_. The red/black dashed lines correspond to the C─H···F interactions or *π*···*π* interactions, respectively. Atom colors: Cu = brown, S = yellow, P = purple, Cl = green, F = chartreuse, C = gray.

It is widely recognized that the interaction between and within clusters plays a crucial role in both crystal growth and stability. Therefore, a detailed elucidation of the weak interactions in Cu_41_ is of great importance. As illustrated in **Figure** **3**A, the benzene rings between adjacent ─SC_6_H_3_F_2_ ligands as well as adjacent ─SC_6_H_3_F_2_ ligands and ─P(PhF)_3_ ligands on the Cu_6_(SC_6_H_3_F_2_)_6_(P(PhF)_3_)_3_ rings in Cu_41_ exhibit a remarkable parallel alignment, resulting in strong intracluster *π*···*π* interactions. Moreover, as depicted in Figure 3B, the unit cell of Cu_41_ consists of four Cu_41_ nanoclusters, arranged in an enantiomeric form. Within the Cu_41_ nanoclusters, multiple C─H···F bonds (2.387–2.899 Å) can be observed. The assembly of clusters in a cell is facilitated by C─H···F hydrogen interactions (2.515–2.649 Å) between ─SC_6_H_3_F_2_ and P(PhF)_3_ ligands. Furthermore, the regioselective arrangement of ─SC_6_H_3_F_2_ and P(PhF)_3_ determines the preferred orientation during the formation of array intercluster assemblies. These intracluster *π*···*π* interactions enable Cu_41_ to maintain stability in the CH_2_Cl_2_ solution for several weeks, as confirmed by ESI data and digital photos (Figure S4, Supporting Information). Furthermore, the thermal stability of [Cu_41_(SC_6_H_3_F_2_)_15_(P(PhF)_3_)_6_Cl_3_H_25_]^2−^ was carefully assessed in both solution and solid states. The nanocluster was dissolved in CHCl_3_ and heated at 50 °C in an oil bath for half an hour. As depicted in Figure S5A (Supporting Information), no signal peak attributable to the [Cu_41_(SC_6_H_3_F_2_)_15_(P(PhF)_3_)_6_Cl_3_H_25_]^2−^ in the ESI–MS spectrum was observed, indicating poor thermal stability of the nanocluster in solution. Subsequently, a thermal stability test was conducted on the solid sample. [Cu_41_(SC_6_H_3_F_2_)_15_(P(PhF)_3_)_6_Cl_3_H_25_]^2−^ stored in a sealed tube was heated for 1 h at 50 °C, and the color of the nanocluster remained unchanged. As shown in Figure S5B (Supporting Information), several peaks corresponding to [Cu_41_(SC_6_H_3_F_2_)_15_(P(PhF)_3_)_6_Cl_5_H_22_CN]^2−^, [Cu_41_(SC_6_H_3_F_2_)_15_(P(PhF)_3_)_5_Cl_5_H_22_CN]^2−^, and [Cu_41_(SC_6_H_3_F_2_)_15_(P(PhF)_3_)_4_Cl_5_H_22_CN]^2−^ were observed, indicating improved stability of the [Cu_41_(SC_6_H_3_F_2_)_15_(P(PhF)_3_)_6_Cl_3_H_25_]^2−^ in the solid state. In the crystal packing, the structures on the left and right are closely combined, leading to intercluster C─H···F─C interactions. Strong intracluster *π*···*π* and multiple C─H···F bond interactions contribute to the enhanced stability of [Cu_41_(SC_6_H_3_F_2_)_15_(P(PhF)_3_)_6_Cl_3_H_25_]^2−^ in the solid state. Overall, the comprehensive analysis of weak interactions in Cu_41_ provides valuable insights into its crystal growth and stability.

**Figure 4:**
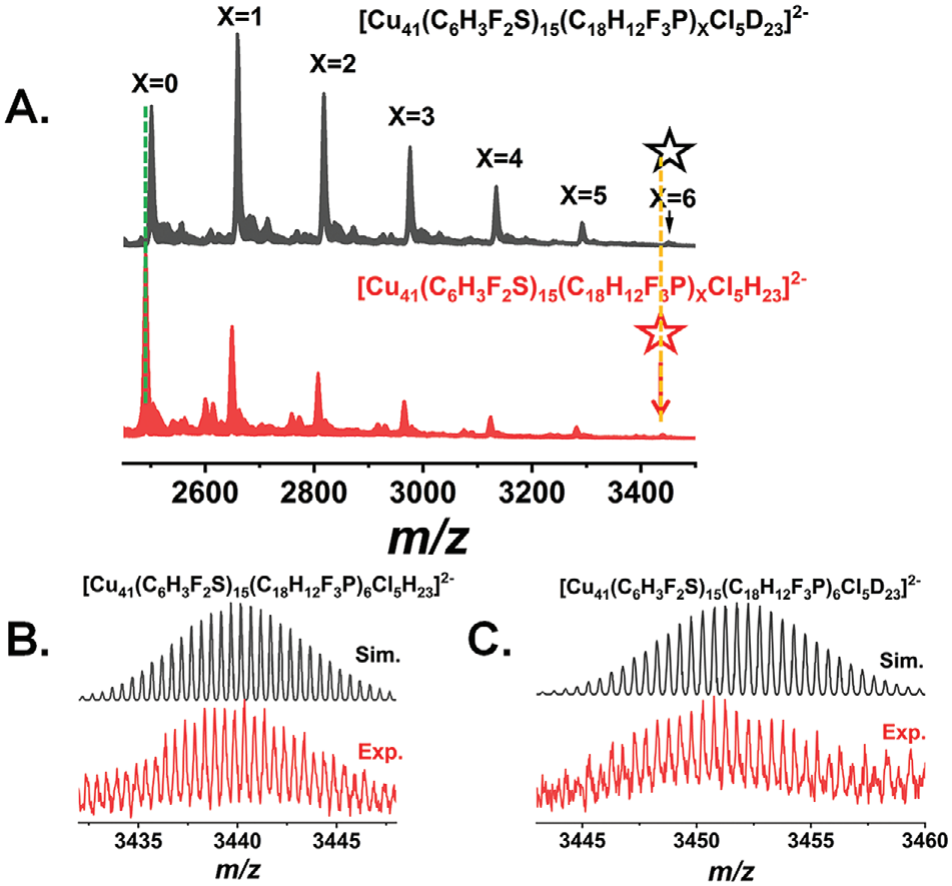
A) ESI–MS data of [Cu_41_(SC_6_H_3_F_2_)_15_Cl_5_(P(PhF)_3_)_x_(H)_23_]^2−^ (x = 0–6) (black trace) and [Cu_41_(SC_6_H_3_F_2_)_15_(P(PhF)_3_)_x_Cl_5_D_23_]^2−^ (x = 0–6) (red trace) in negative‐ion mode. (B and C) Comparison of experimental mass spectra of the [Cu_41_(SC_6_H_3_F_2_)_15_(P(PhF)_3_)_6_Cl_5_H_23_]^2−^ species and [Cu_41_(SC_6_H_3_F_2_)_15_(P(PhF)_3_)_x_Cl_5_D_23_]^2−^ with their simulated ones, respectively.

To accurately determine the molecular formula of the Cu_41_ cluster, particularly the number of hydrides, the electrospray ionization mass spectrometry (ESI–MS) was employed. This technique allowed for a careful assessment of the hydride count and charge state of the nanocluster. As shown in **Figure** **4**, the negative‐mode ESI–MS spectrum showed several peaks with m/z 0.5 separation. A peak with high abundance is observed at *m/z* 2491.38 Da, which can be assigned to [Cu_41_(SC_6_H_3_F_2_)_15_(P(PhF)_3_)_0_Cl_5_H_23_]^2−^ (Cal. 2491.49 Da). In addition, other peaks at 2649.39, 2807.40, 2965.41, 3123.43, 3281.44 and 3439.87 Da were corresponded to [Cu_41_(SC_6_H_3_F_2_)_15_(P(PhF)_3_)_1_Cl_5_H_23_]^2−^ (Cal. 2649.52 Da), [Cu_41_(SC_6_H_3_F_2_)_15_(P(PhF)_3_)_2_Cl_5_H_23_]^2−^ (Cal. 2807.56 Da), [Cu_41_(SC_6_H_3_F_2_)_15_(P(PhF)_3_)_3_Cl_5_H_23_]^2−^ (Cal. 2965.59 Da), [Cu_41_(SC_6_H_3_F_2_)_15_(P(PhF)_3_)_4_Cl_5_H_23_]^2−^ (Cal. 3123.62 Da), [Cu_41_(SC_6_H_3_F_2_)_15_(P(PhF)_3_)_5_Cl_5_H_23_]^2−^ (Cal. 3281.65 Da) and [Cu_41_(SC_6_H_3_F_2_)_15_(P(PhF)_3_)_6_Cl_5_H_23_]^2−^ (Cal. 3439.68 Da), respectively. The calculated isotopic patterns closely matched the experimental ones (Figure S6, Supporting Information). It is worth noting that the loss of phosphine ligands in the ESI–MS spectra has been reported in previous studies.^[^ ^47^, ^48^, ^49^, ^50^, ^51^ ^]^ Furthermore, the Cu_41_ cluster synthesized using NaBD_4_ was also subjected to ESI analysis. As shown in Figure 4, several peaks corresponding to a −2 charge were observed at m/z = 2503.17, 2661.21, 2819.24, 2977.27, 3135.30, 3293.31, 3451.36 Da, with an upshift of m/z 11.6 from [Cu_41_(SC_6_H_3_F_2_)_15_(P(PhF)_3_)_x_Cl_5_H_23_]^2−^ (x = 0–6). These findings were consistent with the single‐crystal X‐ray diffraction (SCXRD) results regarding the Cu atoms, thiol, and phosphine ligands. Since the crystal structure revealed the presence of only three Cl ligands, the ESI–MS results indicated the replacement of two H atoms with two Cl atoms, similar to the ESI–MS data of the Cu_32_ nanoclusters.^[^ ^14^ ^]^ Considering the SCXRD and ESI–MS results, the synthesized copper nanocluster can be identified as [Cu_41_(SC_6_H_3_F_2_)_15_(P(PhF)_3_)_6_Cl_3_H_25_]^2−^. In addition, the ^1^HNMR spectra of [Cu_41_(SC_6_H_3_F_2_)_15_Cl_3_(P(PhF)_3_)_6_(H)_25_]^2−^ and [Cu_41_(SC_6_H_3_F_2_)_15_Cl_3_(P(PhF)_3_)_6_(D)_25_]^2−^ as well as the ^2^HNMR of [Cu_41_(SC_6_H_3_F_2_)_15_Cl_3_(P(PhF)_3_)_6_(D)_25_]^2−^ were meticulously conducted. Additionally, DFT calculations on the hydride chemical shifts of [Cu_41_(SC_6_H_3_F_2_)_15_Cl_3_(P(PhF)_3_)_6_(H)_25_]^2−^ were carried out. As depicted in Figure S7 (Supporting Information), both [Cu_41_(SC_6_H_3_F_2_)_15_Cl_3_(P(PhF)_3_)_6_(H)_25_]^2−^ and [Cu_41_(SC_6_H_3_F_2_)_15_Cl_3_(P(PhF)_3_)_6_(D)_25_]^2−^ nanoclusters exhibited similar peaks in the ^1^HNMR spectra. The peaks observed at 7.5–5.8 ppm correspond to protons in the ─SC_6_H_3_F_2_ and P(PhF)_3_ phenyl groups. The broadening of peaks between 7.5–5.8 ppm was attributed to differences in the ligands' environments. Furthermore, [Cu_41_(SC_6_H_3_F_2_)_15_Cl_3_(P(PhF)_3_)_6_(H)_25_]^2−^ nanoclusters displayed additional peaks centered between 0–1 ppm, 1.5–3.5 ppm, and 3.5–4.7 ppm in the ^1^HNMR spectrum, compared with the [Cu_41_(SC_6_H_3_F_2_)_15_Cl_3_(P(PhF)_3_)_6_(D)_25_]^2−^ nanoclusters (Figure S7A, Supporting Information). Additionally, the ^2^HNMR of [Cu_41_(SC_6_H_3_F_2_)_15_Cl_3_(P(PhF)_3_)_6_(H)_25_]^2−^ revealed peaks centered between 0–1 ppm, 1.5–3.5 ppm, and 4.0–4.7 ppm, corresponding to the hydrides (Figure S7B, Supporting Information). The area ratio is approximately 3:14:8. Moreover, the simulated ^1^HNMR shifts for the optimized structure are shown in Figure S8 (Supporting Information). The hydride chemical shifts of [Cu_41_(SC_6_H_3_F_2_)_15_Cl_3_(P(PhF)_3_)_6_(H)_25_]^2−^ were centered between 2–2.8 ppm, 3.5–5.5 ppm, and 6.0–7.8 ppm, with an area ratio of 8:14:3. The experimental results were in good agreement with the theoretical data, considering the overall deviation. Hence, the structure of [Cu_41_(SC_6_H_3_F_2_)_15_Cl_3_(P(PhF)_3_)_6_(H)_25_]^2−^ is deemed reasonable in light of these data. X‐ray photoelectron spectroscopy (XPS) was conducted to validate the composition and valence state of Cu_41_. As illustrated in Figure S9 (Supporting Information), the Cu 2p1/2 and 2p3/2 peaks at 952.8 and 933 eV, with a doublet separation of 19.8 eV, indicate the presence of Cu(I) (Figure S10, Supporting Information). In addition, the Cu LMM Auger spectrum with a main peak at 570.88 eV confirms that all copper atoms are in the oxidation state of Cu(I), which aligns with the overall composition of Cu_41_ (Figure S11, Supporting Information). In addition, as depicted in Figure S12 (Supporting Information), the thermogravimetric analysis (TGA) of the Cu_41_ cluster, represents its weight loss process when warming up to 600 °C, the total weight reduction is 52.69%, which is basically consistent with the theoretical value (52.86%), and the remaining material is Cu_2_S.

### 2.2 Hydride Positions, Cl→H Replacement and Optical Spectrum by DFT

**Figure 5:**
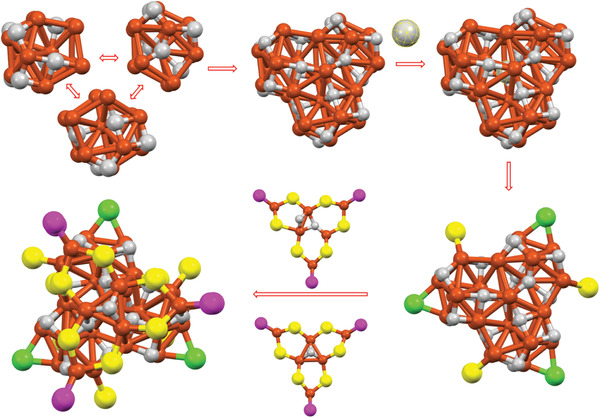
Optimal hydride sites in the [Cu_41_(SR)_15_(PR_3_)_6_Cl_3_H_25_]^2−^.

Density functional theory (DFT) calculations were meticulously conducted to investigate the most probable hydride position of  [Cu_41_(SC_6_H_3_F_2_)_15_(P(PhF)_3_)_6_Cl_3_H_25_]^2−^ and replacement of hydrides with chlorides to yield the [Cu_41_(SC_6_H_3_F_2_)_15_(P(PhF)_3_)_6_Cl_5_H_23_]^2−^ cluster observed in the ESI–MS experiment.^[^ ^52^ ^]^ As depicted in Figure S13 (Supporting Information), two optimized structures were obtained, differing in the presence of H─ in the core assembled by three twisted Cu_13_ units. Both optimized Cu_41_ nanoclusters (Cu_41_^T^) exhibited partial distortion on the shell surface, indicating the reasonableness of the optimized structures. The structure with H^−^ in the core had approximately 0.5 eV lower energy than the structure without H^−^ in the core. Following the principle of minimum energy, we conclude that the structure with H in the core is the most reasonable. Further analysis in **Figure** **5** revealed that 22 hydrides were located on the Cu_29_ core, with seven hydrides on each Cu_13_ unit and one at the core junction. The remaining three hydrides were situated on the shells of Cu_12_(SR)_12_(PR_3_)_6_ on both sides. Notably, two hydride sites were in proximity to the core–ligand interface, suggesting their susceptibility to Cl→H replacement reactions. After the replacement of two H with Cl, the [Cu_41_(SC_6_H_3_F_2_)_15_(P(PhF)_3_)_6_Cl_5_H_23_]^2−^ structure, as shown in Figure S13 (Supporting Information), also exhibited the lowest energy.

**Figure 6:**
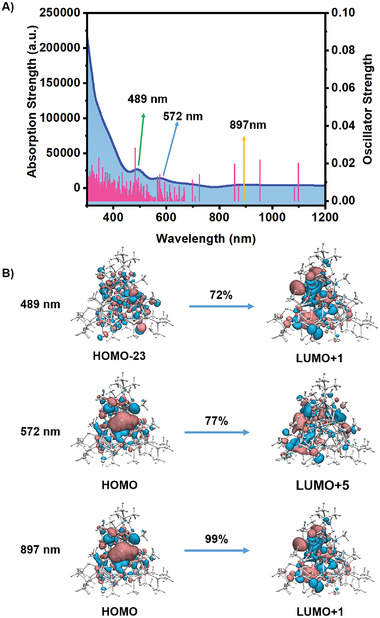
A) The calculated UV–vis absorption spectrum of [Cu_41_(SR)_15_(PR_3_)_6_Cl_3_H_25_]^2−^ and B) the Kohn–Sham molecular orbital diagram.

Furthermore, the UV–vis absorption spectrum was calculated based on the optimized structures, and simplified time‐dependent DFT (sTD‐DFT) calculations were performed using the PBE/def2‐SV(P) method to simulate the optical absorption spectra.^[^ ^53^ ^]^ As shown in **Figure** **6**A, several absorption peaks at 489, 572, and 897 nm were predicted based on the oscillator strength. However, it should be noted that the absorption peaks are not entirely prominent, which is a common phenomenon observed in Cu nanoclusters (Figure S14, Supporting Information). The Kohn–Sham molecular orbitals of the [Cu_41_(SR)_15_(PR_3_)_6_Cl_3_H_25_]^2−^ nanoclusters are illustrated in Figure 6B. The highest occupied molecular orbital (HOMO) and the lowest unoccupied molecular orbital (LUMO) are primarily composed of Cu and S atoms. Specifically, the absorption peak at 489 nm is mainly attributed to the transition from HOMO‐23 to LUMO+1, the absorption peak at 572 nm is mainly attributed to the transition from HOMO to LUMO+5, and the absorption peak at 897 nm is mainly attributed to the transition from HOMO to LUMO+1.

### 2.3 The Nanocluster‐to‐Nanocluster Transformation

**Figure 7:**
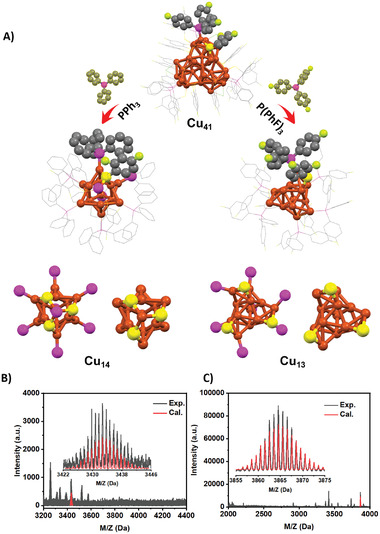
A) the addition of PPh_3_ and P(PhF)_3_ ligands carry out the nanocluster‐to‐nanocluster transformation; the ESI data of (B) [Cu_14_(SC_6_H_3_F_2_)_3_(PPh_3_)_8_H_10_]^+^ and C) [Cu_13_(SC_6_H_3_F_2_)_3_(P(PhF)_3_)_7_H_10_]^0.^

As previously reported, thiol ligands have been demonstrated to effectively regulate structural transformation and construct novel clusters.^[^ ^54^, ^55^, ^56^ ^]^ Similarly, phosphine ligands have also been utilized for structural regulation of clusters.^[^ ^57^, ^58^, ^59^ ^]^ While the structural regulation of gold, silver, and alloy clusters has been extensively studied, there has been limited research on the structural regulation of copper clusters. Interestingly, the addition of PPh_3_ and P(PhF)_3_ ligands induces a nanocluster‐to‐nanocluster transformation. Specifically, PPh_3_ induces [Cu_41_(SC_6_H_3_F_2_)_15_(P(PhF)_3_)_6_Cl_3_H_25_]^2−^ to convert into [Cu_14_(SC_6_H_3_F_2_)_3_(PPh_3_)_8_H_10_]^+^, while P(PhF)_3_ ligands induce [Cu_41_(SC_6_H_3_F_2_)_15_(P(PhF)_3_)_6_Cl_3_H_25_]^2−^ to convert into [Cu_13_(SC_6_H_3_F_2_)_3_(P(PhF)_3_)_7_H_10_]^0^ (**Figure** **7**A; Tables S2 and S3,Supporting Information). These transformations have been confirmed by single‐crystal X‐ray crystallography and ESI–MS spectra.^[^ ^60^ ^]^ The UV–vis spectra of [Cu_13_(SC_6_H_3_F_2_)_3_(P(PhF)_3_)_7_H_10_]^0^ and [Cu_14_(SC_6_H_3_F_2_)_3_(PPh_3_)_8_H_10_]^+^ are shown in Figure S15 (Supporting Information). The [Cu_13_(SC_6_H_3_F_2_)_3_(P(PhF)_3_)_7_H_10_]^0^ exhibited a peak at 388 nm, and the [Cu_14_(SC_6_H_3_F_2_)_3_(PPh_3_)_8_H_10_]^+^ showed a peak at 428 nm. The overall structures of [Cu_14_(SC_6_H_3_F_2_)_3_(PPh_3_)_8_H_10_]^+^ and [Cu_13_(SC_6_H_3_F_2_)_3_(P(PhF)_3_)_7_H_10_]^0^ were depicted in Figures S16A and S17A (Supporting Information), respectively. The chiral arrangement of peripheral achiral ligands leads to the presence of enantiomers in the cluster cell. However, it is worth noting that the metal core of [Cu_13_(SC_6_H_3_F_2_)_3_(P(PhF)_3_)_7_H_10_]^0^ is chiral due to structural distortion, while the metal core of [Cu_14_(SC_6_H_3_F_2_)_3_(PPh_3_)_8_H_10_]^+^ is achiral due to its high symmetry (Figures S16B and S17B, Supporting Information). The structure of [Cu_13_(SC_6_H_3_F_2_)_3_(P(PhF)_3_)_7_H_10_]^0^ beared resemblance to Cu_13_H_10_(SC_6_H_3_FCl)_3_(PPh_3_)_7_, as reported by Huang group.^[^ ^61^ ^]^ The total crystal structure of [Cu_13_(SC_6_H_3_F_2_)_3_(P(PhF)_3_)_7_H_10_]^0^ nanocluster also exhibited a threefold symmetry (C_3_) axis. Additionally, [Cu_13_(SC_6_H_3_F_2_)_3_(P(PhF)_3_)_7_H_10_]° can be viewed as an assembly of three Cu_4_ tetrahedrons protected by two phosphine ligands and one thiol ligand, further capped by one CuPPh_3_ terminal staple (Figure S18, Supporting Information). Similarly, [Cu_14_(SC_6_H_3_F_2_)_3_(PPh_3_)_8_)_3_H_10_]^+^ can be viewed as an assembly of three Cu_4_ clusters protected by two phosphine ligands and one thiol ligand, further capped by two CuPPh_3_ terminal staples.^[^ ^62^, ^63^ ^]^ In contrast, the Cu_4_ structure in [Cu_14_(SC_6_H_3_F_2_)_3_(PPh_3_)_8_)_3_H_10_]^+^ exhibits a V‐shaped configuration rather than a tetrahedral structure (Figure S18, Supporting Information). The structure of the Cu_13_ core in [Cu_14_(SC_6_H_3_F_2_)_3_(PPh_3_)_8_H_10_]^+^ clusters bears similarities to that of [Cu_13_(SC_6_H_3_F_2_)_3_(P(PhF)_3_)_7_H_10_]^0^ nanoclusters, but it undergoes a structural distortion. This distortion is attributed to the formation of a Cu(SR)_3_(PR_3_) motif, where a CuPPh_3_ is attached to the three thiol ligands in [Cu_14_(SC_6_H_3_F_2_)_3_(PPh_3_)_8_H_10_]^+^. In comparison to the three thiol ligands in the [Cu_13_(SC_6_H_3_F_2_)_3_(P(PhF)_3_)_7_H_10_]^0^ nanocluster, the positions of the three thiol ligands in [Cu_14_(SC_6_H_3_F_2_)_3_(PPh_3_)_8_H_10_]^+^ are more concentrated along the C_3_ axis, resulting in a distorted core structure.The ESI–MS measure was meticulously performed,  revealing a peak with high abundance at m/z 3865.20 Da in positive mode. This peak can be assigned to [Cu_13_(SC_6_H_3_F_2_)_3_(P(PhF)_3_)_7_H_10_]+CuP(PhF)_3_]^+^ (Cal. 3865.16 Da), confirming the accurate formula of [Cu_13_(SC_6_H_3_F_2_)_3_(P(PhF)_3_)_7_H_10_]^0^ ((Figure 7C). Additionally, a peak is observed at m/z 3433.82 Da, corresponding to [Cu_14_(SC_6_H_3_F_2_)_3_(PPh_3_)_8_H_10_]^+^ (Cal. 3433.85 Da), confirming the correct formula of [Cu_14_(SC_6_H_3_F_2_)_3_(PPh_3_)_8_H_10_]^+^ (Figure 7B). Figure S19 (Supporting Information) illustrates the simulated hydride sites in the optimized structure of [Cu_13_(SC_6_H_3_F_2_)_3_(P(PhF)_3_)_7_H_10_]^0^ nanoclusters. Each of the 10 hydrides is linked to three Cu atoms in the µ_3_ mode, and the hydride types can be divided into four groups based on the Cu_3_ triangular coordination environment. The distribution of hydrides is as follows: 1 at the top, 3+3 in the middle, and 3 at the bottom. Specifically, the top H‐4 binds to the three SR ligands and the Cu_3_ triangle (Figure S19g, Supporting Information). In the middle, the six hydrides are divided into two groups, where three H‐1/2/3 hydrides are attached to a Cu_3_ triangle with one SR ligand and one P(PhF)_3_ ligand (Figure S19h, Supporting Information), and the remaining three H‐5/6/7 hydrides are attached to a Cu_3_ triangle with two P(PhF)_3_ (Figure S19i, Supporting Information). Finally, at the bottom H‐8/9/10 three hydrides are connected to a Cu_3_ triangle with one SR ligand and two P(PhF)_3_ ligands (Figure S19j, Supporting Information). Similarly, the simulated hydride sites in the optimized structure of [Cu_14_(SC_6_H_3_F_2_)_3_(PPh_3_)_8_H_10_]^+^ nanoclusters are shown in Figure S20 (Supporting Information). The bonding modes of hydrides are also divided into four types: µ_3_ (3+3), µ_4_ (3), and µ_5_ (1). H‐10 binds to the Cu_5_ unit with three SR ligands in a bonding pattern of µ_5_ (Figure S20g, Supporting Information). The hydrides bonded in µ_3_ mode are divided into two groups, where H‐1/3/5 (Figure S20h, Supporting Information) binds to Cu_3_ with one TPP and SR ligand, and H‐7/8/9 (Figure S20j, Supporting Information) binds to Cu_3_ with two TPP ligands. Finally, the remaining H‐2,4,6 three hydrides are connected to the Cu_4_ unit with one SR ligand and two TPP (Figure S20i, Supporting Information).

Furthermore, the investigation into the conversation between [Cu_14_(SC_6_H_3_F_2_)_3_(PPh_3_)_8_H_10_]^+^ to [Cu_13_(SC_6_H_3_F_2_)_3_(P(PhF)_3_)_7_H_10_]^0^ was conducted meticulously (Figures S21 and S22, Supporting Information). Initially, 20 mg of TPP was slowly introduced into a CH_2_Cl_2_ solution containing 5 mg of [Cu_13_(SC_6_H_3_F_2_)_3_(P(PhF)_3_)_7_H_10_]^0^. Subsequently, time‐dependent UV–vis spectra and ESI–MS spectra tracking were performed. As depicted in Figure S21A (Supporting Information), the UV–vis absorption signal peak of [Cu_13_(SC_6_H_3_F_2_)_3_(P(PhF)_3_)_7_H_10_]^0^ at 388 nm gradually diminished over time, eventually displaying a weak shoulder UV–vis absorption at 426 nm. Analysis of the ESI–MS spectra revealed that the signal peak of [Cu_14_(SC_6_H_3_F_2_)_3_(PPh_3_)_8_H_10_]^+^ emerged shortly after the initiation of the reaction. Simultaneously, a signal peak of [Cu_14_(SC_6_H_3_F_2_)_3_(PPh_3_)_7_((P(PhF)_3_)_1_)H_10_]^+^ was also observed (Figure S21B, Supporting Information). The calculated and experimental isotope distribution patterns of [Cu_14_(SC_6_H_3_F_2_)_3_(PPh_3_)_8_H_10_]^+^ and [Cu_14_(SC_6_H_3_F_2_)_3_(PPh_3_)_7_((P(PhF)_3_)_1_)H_10_]^+^ exhibited good agreement (Figure S21C,D, Supporting Information). Subsequently, after 120 minutes of reaction, the signal peaks of Cu_14_(SC_6_H_3_F_2_)_3_(PPh_3_)_8_)_3_H_10_]^+^ and [Cu_14_(SC_6_H_3_F_2_)_3_(PPh_3_)_7_((P(PhF)_3_)_1_)H_10_]^+^ were still observed, with the signal intensity of [Cu_14_(SC_6_H_3_F_2_)_3_(PPh_3_)_8_H_10_]^+^ being stronger than that of [Cu_14_(SC_6_H_3_F_2_)_3_(PPh_3_)_7_((P(PhF)_3_)_1_)H_10_]^+^. These results indicate that [Cu_13_(SC_6_H_3_F_2_)_3_(P(PhF)_3_)_7_H_10_]° can also be transformed into [Cu_14_(SC_6_H_3_F_2_)_3_(PPh_3_)_8_H_10_]^+^ under the influence of TPP, accompanied by incomplete replacement of [Cu_14_(SC_6_H_3_F_2_)_3_(PPh_3_)_7_((P(PhF)_3_)_1_)H_10_]^+^. Additionally, 20 mg of P(PhF)_3_ was slowly added into the CH_2_Cl_2_ solution of 5 mg of Cu_14_(SC_6_H_3_F_2_)_3_(PPh_3_)_8_H_10_]^+^, and subsequent time‐dependent UV–vis spectra and ESI–MS spectra tracking were also performed. As shown in Figure S22A (Supporting Information), the UV–vis absorption signal peak of Cu_14_(SC_6_H_3_F_2_)_3_(PPh_3_)_8_H_10_]^+^ at 428 nm remained in a similar position within 120 min. Analysis of the ESI–MS spectra revealed a new signal peak of [Cu_14_(SC_6_H_3_F_2_)_3_(PPh_3_)_7_((P(PhF)_3_)_1_)H_10_]^+^ after 15 min of reaction (Figure S22B, Supporting Information), indicating the replacement of one PPh_3_ ligand in the [Cu_14_(SC_6_H_3_F_2_)_3_(PPh_3_)_8_H_10_]^+^ by P(PhF)_3_ ligands. The calculated and experimental isotope distribution patterns of [Cu_14_(SC_6_H_3_F_2_)_3_(PPh_3_)_8_H_10_]^+^ and [Cu_14_(SC_6_H_3_F_2_)_3_(PPh_3_)_7_((P(PhF)_3_)_1_)H_10_]^+^ exhibited good agreement (Figure S22C,D, Supporting Information). After 120 min, the signal of [Cu_14_(SC_6_H_3_F_2_)_3_(PPh_3_)_8_H_10_]^+^ almost disappeared, and an obvious signal of [Cu_14_(SC_6_H_3_F_2_)_3_(PPh_3_)_7_((P(PhF)_3_)_1_)H_10_]^+^ was observed. This indicated that [Cu_14_(SC_6_H_3_F_2_)_3_(PPh_3_)_8_H_10_]^+^ could not be transformed into [Cu_13_(SC_6_H_3_F_2_)_3_(P(PhF)_3_)_7_H_10_]^0^ under the influence of P(PhF)_3_, while mainly ligand exchange occurred. This difference in transformation indicates that structural integrity has an effect on ligand‐induced structural transformation. These data suggest that the introduction of phosphine ligands can induce a nanocluster‐to‐nanocluster transformation, where the phosphine and thiol ligands co‐protect the copper nanoclusters. This discovery provides a novel synthesis strategy for regulating the construction of copper nanoclusters.

### 2.4 Catalytic Hydrogenation of p‐nitrophenol (p‐NP)

**Figure 8:**
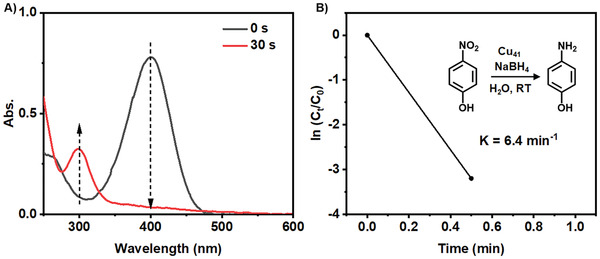
A) The UV–vis absorption spectra of the catalytic solution as a function of time. (B) Plot of ln(C_t_/C_0_) versus time for p‐NP reduction by NaBH_4_ aided with catalyst Cu_41_(SC_6_H_3_F_2_)_15_(P(PhF)_3_)_6_Cl_3_H_25_]^2−^.

Furthermore, we selected the catalytic hydrogenation of p‐nitrophenol (p‐NP) into p‐aminophenol (p‐AP) using BH_4_^−^ as a model reaction to evaluate the catalytic activity of [Cu_41_(SC_6_H_3_F_2_)_15_(P(PhF)_3_)_6_Cl_3_H_25_]^2−^.^[^ ^6^, ^17^, ^45^, ^64^, ^65^ ^]^ The progress of the reduction process using [Cu_41_(SC_6_H_3_F_2_)_15_(P(PhF)_3_)_6_Cl_3_H_25_]^2−^ as the catalyst was conveniently monitored by time‐dependent UV–vis spectroscopy. As shown in **Figure** **8** and Figure S23 (Supporting Information), during the catalytic reaction, the intense absorption peak at 400 nm (p‐NP‐) rapidly decreases within 30 seconds, while a new peak at 300 nm (p‐AP‐) emerges, accompanied by the fading of the solution from bright yellow to colorless (Figure 8A). The decrease in UV–vis absorption at a wavelength of 400 nm can be approximated as a pseudo‐first‐order reaction with a rate constant of k = 6.4 min^−1^ (Figure 8B). The catalytic efficiency of [Cu_41_(SC_6_H_3_F_2_)_15_(P(PhF)_3_)_6_Cl_3_H_25_]^2−^ was significantly higher than that of other copper hydride catalysts, such as Cu_11_H_3_(Tf‐dpf)_6_(OAc)_2_ (Cu_11_H_3_), which achieved complete conversion of p‐NP to p‐AP in 10 min with k = 0.5 min^−1^, and [Cu_57_H_20_(PET)_36_(TPP)_4_]^+^ in 20 min with k = 0.18 min^−1^. This indicates that [Cu_41_(SC_6_H_3_F_2_)_15_(P(PhF)_3_)_6_Cl_3_H_25_]^2−^ exhibits excellent catalytic hydrogenation activity. In comparison, a blank experiment without [Cu_41_(SC_6_H_3_F_2_)_15_(P(PhF)_3_)_6_Cl_3_H_25_]^2−^showed no occurrence of the p‐NP reduction reaction, as confirmed by the unchanged absorption spectrum over 60 minutes when only NaBH_4_ was added (Figure S24, Supporting Information).

## 3 Conclusion

In summary, we have synthesized and characterized a copper‐hydride nanocluster with the formula of [Cu_41_(SC_6_H_3_F_2_)_15_(P(PhF)_3_)_6_Cl_3_H_25_]^2−^. This core–shell nanocluster features a unique assembly where the Cu_29_ kernel is formed by three twisted Cu_13_ units through Cu_4_ face sharing, a rare phenomenon in copper nanoclusters. The theoretical calculations indicate that the hydride atoms are predominantly concentrated on the Cu_29_ kernel, with two hydride sites near the core–ligand interface potentially undergoing Cl→H replacement reactions. The remarkable stability of [Cu_41_(SC_6_H_3_F_2_)_15_(P(PhF)_3_)_6_Cl_3_H_25_]^2−^ is attributed to strong intracluster *π*···*π* interactions and intercluster C─H···F interactions. This stability allows the nanocluster to remain stable in solution for up to one week and the solid state for half a year, making it highly promising for future applications. Additionally, [Cu_41_(SC_6_H_3_F_2_)_15_(P(PhF)_3_)_6_Cl_3_H_25_]^2−^ exhibits excellent catalytic activity as a heterogeneous catalyst, with a high reaction rate (k = 6.4 min^−1^) for the reduction of p‐NP to p‐AP using NaBH_4_. Furthermore, the introduction of additional PPh_3_ and P(PhF)_3_ ligands triggers a nanocluster‐to‐nanocluster transformation in [Cu_41_(SC_6_H_3_F_2_)_15_(P(PhF)_3_)_6_Cl_3_H_25_]^2−^, resulting in the formation of two distinct nanoclusters: [Cu_14_(SC_6_H_3_F_2_)_3_(PPh_3_)_8_H_10_]^+^ and [Cu_13_(SC_6_H_3_F_2_)_3_(P(PhF)_3_)_7_H_10_]^0^. This [Cu_41_(SC_6_H_3_F_2_)_15_(P(PhF)_3_)_6_Cl_3_H_25_]^2−^ provides valuable insights into the assembly of M_13_ units in coinage metal nanoclusters and offers new possibilities for exploring the co‐protection of copper nanoclusters by phosphine and thiol ligands.

## Conflict of Interest

The authors declare no conflict of interest.

## Supporting information

Supporting Information

Supporting Information

## Data Availability

The data that support the findings of this study are available in the supplementary material of this article.
