# Supplementary material for: Total Structure, Structural Transformation and Catalytic Hydrogenation of [Cu41(SC6H3F2)15Cl3(P(PhF)3)6(H)25]2− Constructed from Twisted Cu13 Units
Source: Adv Sci (Weinh). 2023 Dec 8;11(7):2307085. doi: 10.1002/advs.202307085 (PMC10870033; doi:10.1002/advs.202307085)
Supplement: Supplementary file 1 — Supporting Information [file ADVS-11-2307085-s001.pdf]

ADVANCED SCIENCE
Open Access

Supporting Information

for *Adv. Sci.*, DOI 10.1002/advs.202307085

Total Structure, Structural Transformation and Catalytic Hydrogenation of $[Cu_{41}(SC_6H_3F_2)_{15}Cl_3(P(PhF)_3)_6(H)_{25}]^{2-}$ Constructed from Twisted Cu_{13} Units

Huimin Zhou, Tengfei Duan, Zidong Lin, Tao Yang, Huijuan Deng, Shan Jin, Yong Pei* and Manzhou Zhu**

Supporting Information

Total Structure, Structural transformation and Catalytic Hydrogenation of $[Cu_{41}(SC_6H_3F_2)_{15}Cl_3(P(PhF)_3)_6(H)_{25}]^{2-}$ Constructed from Twisted Cu_{13} Units

*Huimin Zhou,*ǂ *Tengfei Duan,*ǂ *Zidong Lin,*ǂ *Tao Yang, Huijuan Deng, Shan Jin,* Yong Pei,* Manzhou Zhu**

Experimental Section

Materials

Copper (II) tifluoroacetate hydrate, Copper powder, Tris(4-fluorophenyl)phosphine ($P(PhF)_3$), Triphenylphosphine (PPh_3), 2,4-Difluorobenzenethiol ($HSC_6H_3F_2$), sodium borohydride ($NaBH_4$), sodium borodeuteride ($NaBD_4$, 98 at. % D, 90% [CP]), and high-performance liquid chromatography (HPLC) grade solvents (acetonitrile, chloroform, methanol and hexane) were purchased from TEDIA. All chemicals were used directly without further purification.

Synthesis of $[Cu_{41}(SC_6H_3F_2)_{15}Cl_3(P(PhF)_3)_6(H)_{25}]^{2-}$ Nanocluster

First, 135 mg of Copper (II) tifluoroacetate hydrate and 30 mg of Copper powder were dissolved in a solvent mixture of acetonitrile (5 ml) and chloroform (5 ml). Then, 95 mg Tris(4-fluorophenyl)phosphine and 25 μL of 2,4-Difluorobenzenethiol was added. After 20 min, a freshly prepared solution of $NaBH_4$ (20 mg, dissolved in 2 mL MeOH) was added dropwise to the abovementioned solution to form a red solution immediately. The reaction continued for 5 h at room temperature. The crude product was obtained by rotary evaporation. Then, it was dissolved with dichloromethane and acetonitrile respectively, and the supernatant was collected by centrifugation for purification (5 min at~10,000 rpm). The crystals were crystallized from CH_2Cl_2/hexane at room temperature after 4-6 days and yielded dark red crystals. The yield of $[Cu_{41}(SC_6H_3F_2)_{15}Cl_3(P(PhF)_3)_6(H)_{25}]^{2-}$ (15 mg, 0.0022 mmol) was about 9.5% (Cu atom basis).

In a series of studies, the use of dual ligands in the synthesis of copper nanoclusters has proven beneficial for enhancing their stability. Consequently, thiol and phosphine ligands were specifically chosen to facilitate the synthesis in this research. Notably, tris(4-fluorophenyl)phosphine ($P(PhF)_3$) and 2,4-difluorobenzenethiol ($HSC_6H_3F_2$), with their benzene rings and electron-absorbing F groups, play a crucial role in dispersing electrons on copper cores during cluster construction, leading to the formation of stable copper clusters. Moreover, the selected ligands contribute to the formation of intramolecular π···π interactions and intermolecular C-H···F bonds within the stacked cluster structure, thereby enhancing cluster stability and opening up possibilities for future applications. Hence, the choice of tris(4-fluorophenyl)phosphine ($P(PhF)_3$) and 2,4-difluorobenzenethiol ($HSC_6H_3F_2$) for the synthesis was deliberate and strategic.

Synthesis of $[Cu_{41}(SC_6H_3F_2)_{15}Cl_3(P(PhF)_3)_6(D)_{25}]^{2-}$ Nanocluster

The synthesis procedure of $[Cu_{41}(SC_6H_3F_2)_{15}Cl_3(P(PhF)_3)_6(D)_{25}]^{2-}$ is identical to that of $[Cu_{41}(SC_6H_3F_2)_{15}Cl_3(P(PhF)_3)_6(H)_{25}]^{2-}$ except that $NaBD_4$ was used instead of $NaBH_4$. A high-quality red crystal of $[Cu_{41}(SC_6H_3F_2)_{15}Cl_3(P(PhF)_3)_6(D)_{25}]^{2-}$ was obtained in a solvent mixture of chloroform/hexane within one week for ESI measure.

The nanocluster-to-nanocluster transformation from $[Cu_{41}(SR)_{15}(PR_3)_6Cl_3H_{25}]^{2-}$ to $[Cu_{14}(SC_6H_3F_2)_3(PPh_3)_8H_{10}]^{+}$ and $[Cu_{13}(SC_6H_3F_2)_3(P(PhF)_3)_7H_{10}]^{0}$

To achieve structural transformation of clusters, 10 mg $[Cu_{41}(SR)_{15}(PR_3)_6Cl_3H_{25}]^{2-}$ was dissolved in 5ml CH_2Cl_2, and 20 mg $P(PhF)_3$ ligands was added into the CH_2Cl_2 solution. The reaction lasted for one minute, and 20 ml n-hexane was added to the solution to terminate the experimental reaction. The $[Cu_{13}(SC_6H_3F_2)_3(P(PhF)_3)_7H_{10}]^{0}$ product was collected by centrifugation for purification (5 min at~10,000 rpm). The crystals were crystallized from CH_2Cl_2/hexane at room temperature after 4-6 days and yielded orange crystals with a yield of 15% (0.00069 mmol, 2.40 mg) on the basis of $[Cu_{41}(SC_6H_3F_2)_{15}Cl_3(P(PhF)_3)_6(H)_{25}]^{2-}$. In contrast, by adding PPh_3 ligands to the $[Cu_{41}(SR)_{15}(PR_3)_6Cl_3H_{25}]^{2-}$ solution, the yellow-green crystal crystals $[Cu_{14}(SC_6H_3F_2)_3(PPh_3)_8H_{10}]^{+}$ crystallized from CH_2Cl_2/hexane at room temperature after one week, with a yield of 13% (0.00060 mmol, 1.92 mg) on the basis of $[Cu_{41}(SC_6H_3F_2)_{15}Cl_3(P(PhF)_3)_6(H)_{25}]^{2-}$ was obtained.

Catalytic hydrogenation of p-nitrophenol (p-NP)

According to the previous literatures, hydrogenation of p-nitrophenol (p-NP) was chosen as a probe reaction to investigate the catalytic activity of Cu_{41}. In a typical experiment, Cu_{41} nanocluster (2 mg) was added into the water solution of p-NP (3 ml, 15 mg/L), and the mixture was stirred for 10 min at room temperature. Time-dependent UV-vis absorption spectra were taken after the addition of $NaBH_4$ solid (50 mg, 1.3 mmol). The progress of the reaction was probed by monitoring the change in intensity of p-NP peak at 400 nm as a function of time.

Characterization

All nanoclusters' UV/Vis absorption spectra are recorded using a METASH UV-8000PC. Electrospray ionization time-of-flight mass spectrometry (ESI-TOF-MS) measurement was performed using a UPLC H-class/XEV0G2-XS QTOF high-resolution mass spectrometer. The sample was directly infused into the chamber at 5 μL/min. X-ray photoelectron spectroscopy (XPS) measurements were performed using a Thermo ESCALAB 250 configured with a monochromated Al Kα (1486.8 eV) 150 W X-ray source, 0.5m mm circular spot size, a flood gun to counter charging effects, and an analysis chamber base pressure lower than 1×10^{-9} mbar, and the data were collected with FAT = 20 eV. Thermogravimetric analysis (TGA) (~15 mg sample used) was conducted in a N_2 atmosphereon a TG/DTA 6300 analyzer (Seiko Instruments, Inc), and the heating rate was 10 ^{o}C min^{-1}.

Computational Methods

The density functional theory (DFT) calculations were performed using the Dmol^3 8.0 package,[S1-S3] and the structure of clusters was optimized using the Perdew-Burke-Ernzerhof (PBE) function of generalized gradient approximation (GGA). The DFT-based relativistic semicore pseudopotential (DSPP) and double-numerical plus d-function (DND) basis sets were used for the Cu atoms and the light elements S, C, H, P and Cl, respectively. All calculations were done with a charge state of 2- for Cu_{41}. The convergence criteria of the geometric optimization was set to 1.0×10^{-5} hartree for the energy change, 4.0×10^{-3} hartree per Å for the gradient and 5.0×10^{-3} Å for the displacement. The smearing parameter was set to 0.003 Hartree during geometric optimization.

Based on the optimized structures, the UV-vis absorption spectrum are calculated using the ORCA5.0.2 packages.[S4] Simplified time-dependent DFT (sTD-DFT) calculations use PBE/def2-SV(P) method to simulate optical absorption spectra. In all calculation, the phenyl rings in the clusters have been replaced by CH_3 for computing efficiency. The Molecular orbitals were constructed using Visual Molecular Dynamics (VMD).

All proton chemical shift calculations were carried out using Gaussian 09[S5] at revTPSS/pcSseg-1 level of theory. And Chemical shifts were determined from chemical shielding constants relative to TMS calculated at the same level of theory.

[S1] B. Delley, J. Chem. Phys, 1990, 92, 508-517.
[S2] B. Delley, J. Chem. Phys, 2000, 113, 7756-7764.
[S3] J. P. Perdew and M. Levy, Phys. Rev. Lett., 1983, 51, 1884.
[S4] Neese, F. J. W. I. R. C. M. S., The ORCA program system. 2012, 2 , 73-78.
[S5] Frisch, M. J.; Trucks, G. W.; Schlegel, H. B.; Scuseria, G. E.; Robb, M. A.; Cheeseman, J. R.; Scalmani, G.; Barone, V.; Petersson, G. A.; Nakatsuji, H.; Li, X.; Caricato, M.; Marenich, A.; Bloino, J.; Janesko, B. G.; Gomperts, R.; Mennucci, B.; Hratchian, H. P.; Ortiz, J. V.; Izmaylov, A. F.; Sonnenberg, J. L.; Williams-Young, D.; Ding, F.; Lipparini, F.; Egidi, F.; Goings, J.; Peng, B.; Petrone, A.; Henderson, T.; Ranasinghe, D.; Zakrzewski, V. G.; Gao, J.; Rega, N.; Zheng, G.; Liang, W.; Hada, M.; Ehara, M.; Toyota, K.; Fukuda, R.; Hasegawa, J.; Ishida, M.; Nakajima, T.; Honda, Y.; Kitao, O.; Nakai, H.; Vreven, T.; Throssell, K.; J. A. Montgomery, J; Peralta, J. E.; Ogliaro, F.; Bearpark, M.; Heyd, J. J.; Brothers, E.; Kudin, K. N.; Staroverov, V. N.; Keith, T.; Kobayashi, R.; Normand, J.; Raghavachari, K.; Rendell, A.; Burant, J. C.; Iyengar, S. S.; Tomasi, J.; Cossi, M.; Millam, J. M.; Klene, M.; Adamo, C.; Cammi, R.; Ochterski, J. W.; Martin, R. L.; Morokuma, K.; Farkas, O.; Foresman, J. B.; Fox, D. J. Gaussian 09, Revision D.01; Gaussian: Wallingford, CT, USA, 2013.

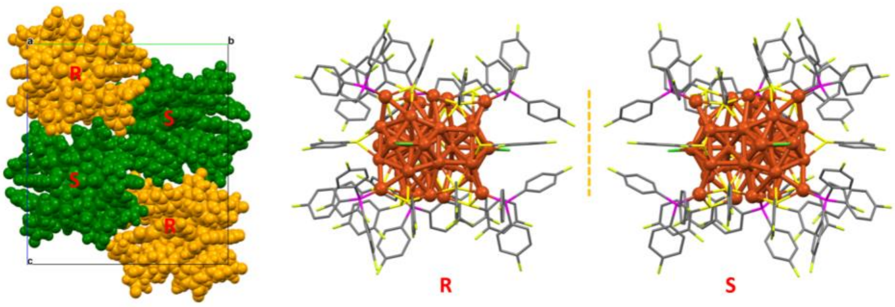

Figure S1. Enantiomers crystallized as a racemic mixture (in a ratio of 1:1).

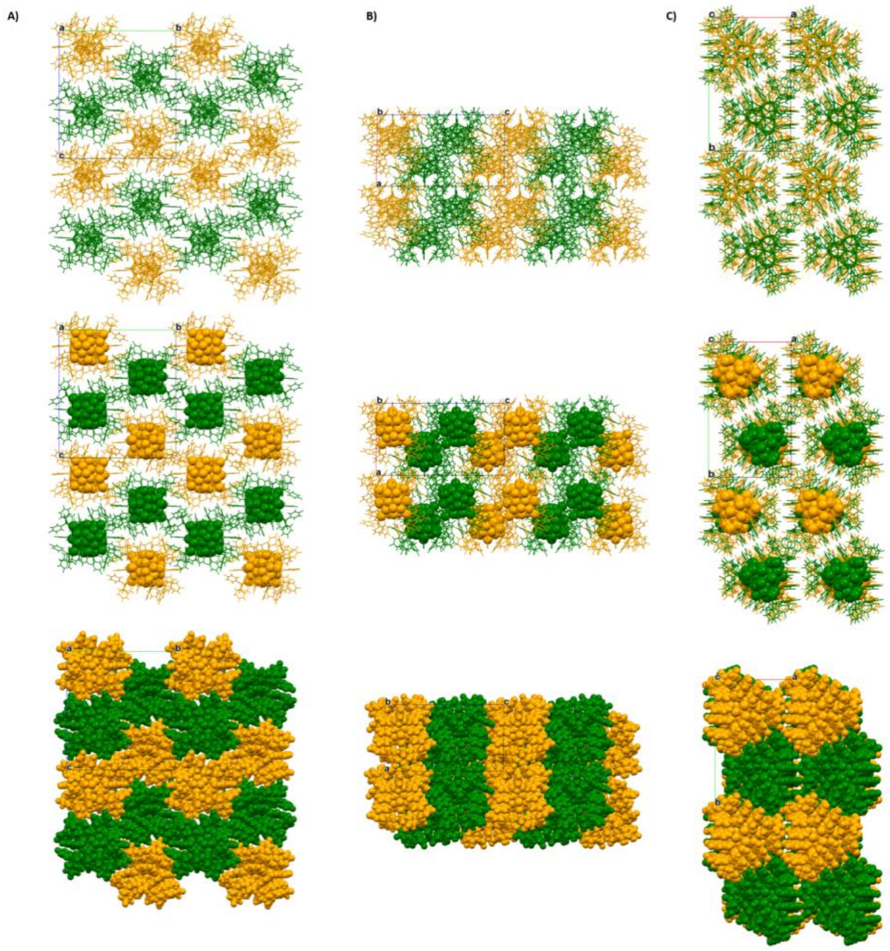

Figure S2. The packing mode of $[Cu_{41}(SC_6H_3F_2)_{15}Cl_3(P(PhF)_3)_6(H)_{25}]^{2-}$ defaulting from a, b and c.

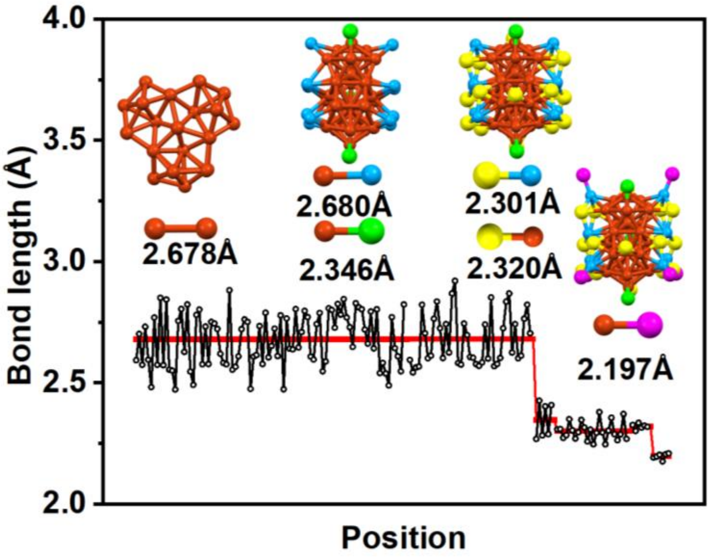

Figure S3. The Bond lengths of Cu_{41} nanoclusters, the numbers marked in the figure are the average key lengths. Color label: grown= Cu_{kernel}; blue=Cu_{shell} ; yellow=S; purple=P; green=Cl.

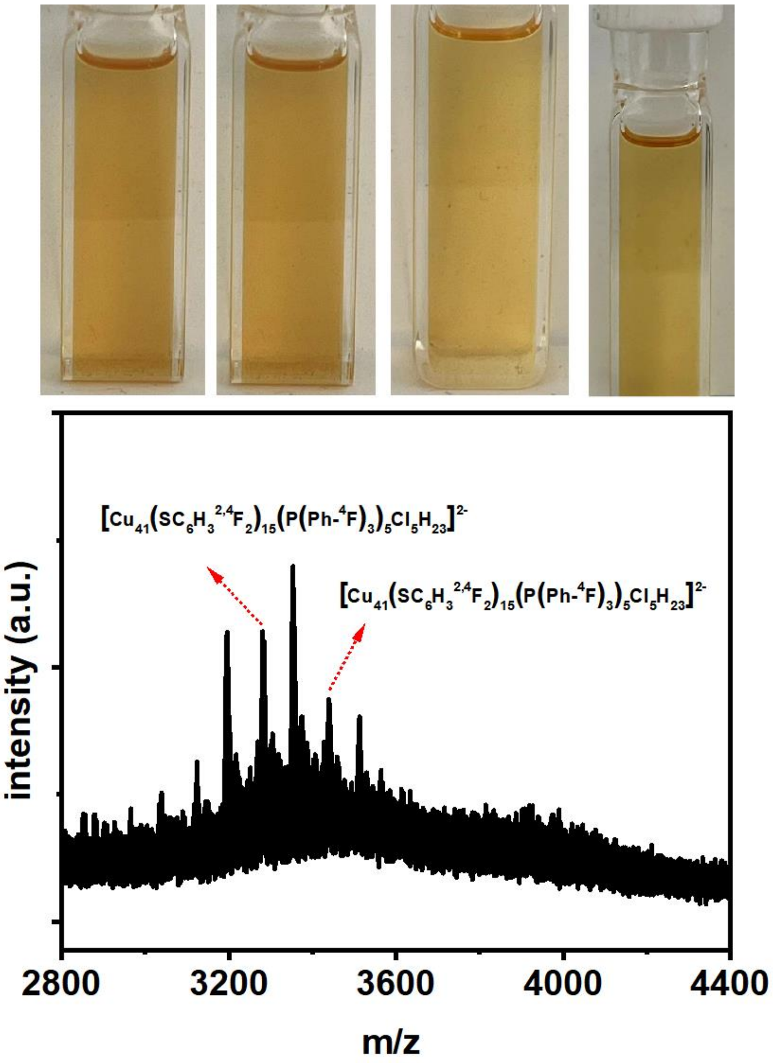

Figure S4. Digital photos of $[Cu_{41}(SC_6H_3F_2)_{15}Cl_3(P(PhF)_3)_6(H)_{25}]^{2-}$ and the ESI data from the sample four days later in room temperature.

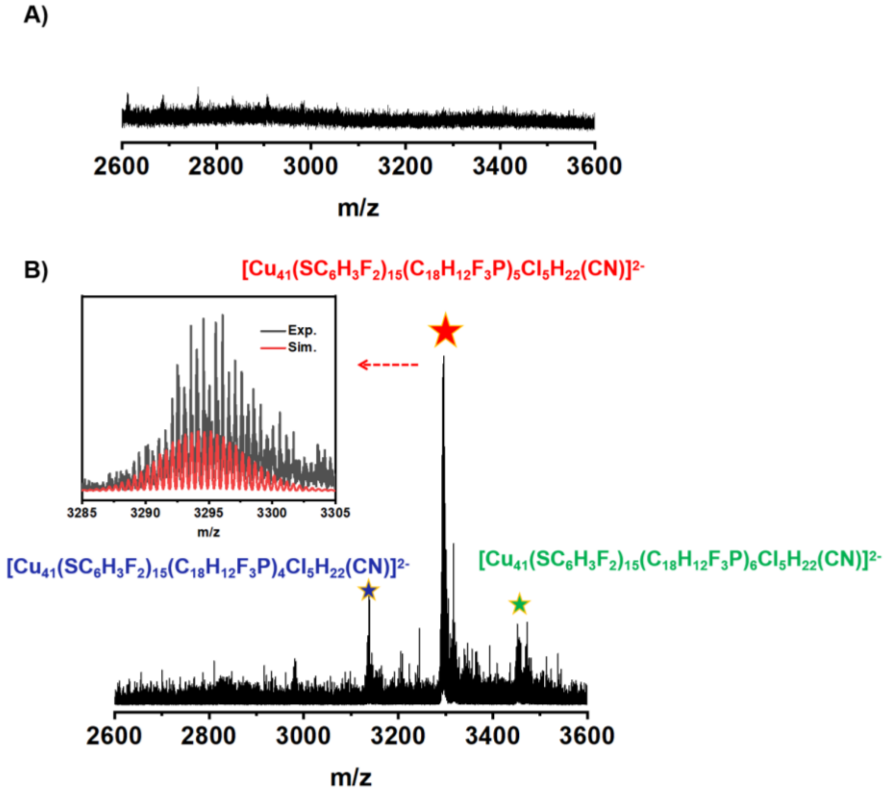

Figure S5. The ESI-MS data of $[Cu_{41}(SC_6H_3F_2)_{15}(P(PhF)_3)_6Cl_3H_{25}]^{2-}$ after thermodynamic stability test. A) The ESI-MS data of $[Cu_{41}(SC_6H_3F_2)_{15}(P(PhF)_3)_6Cl_3H_{25}]^{2-}$ dissolved in $CHCl_3$ and heated at 50°C in oil bath for half an hour. B) The ESI-MS data of $[Cu_{41}(SC_6H_3F_2)_{15}(P(PhF)_3)_6Cl_3H_{25}]^{2-}$ stored in a shrek tube was heated for 1 h under 50 ^{o}C.

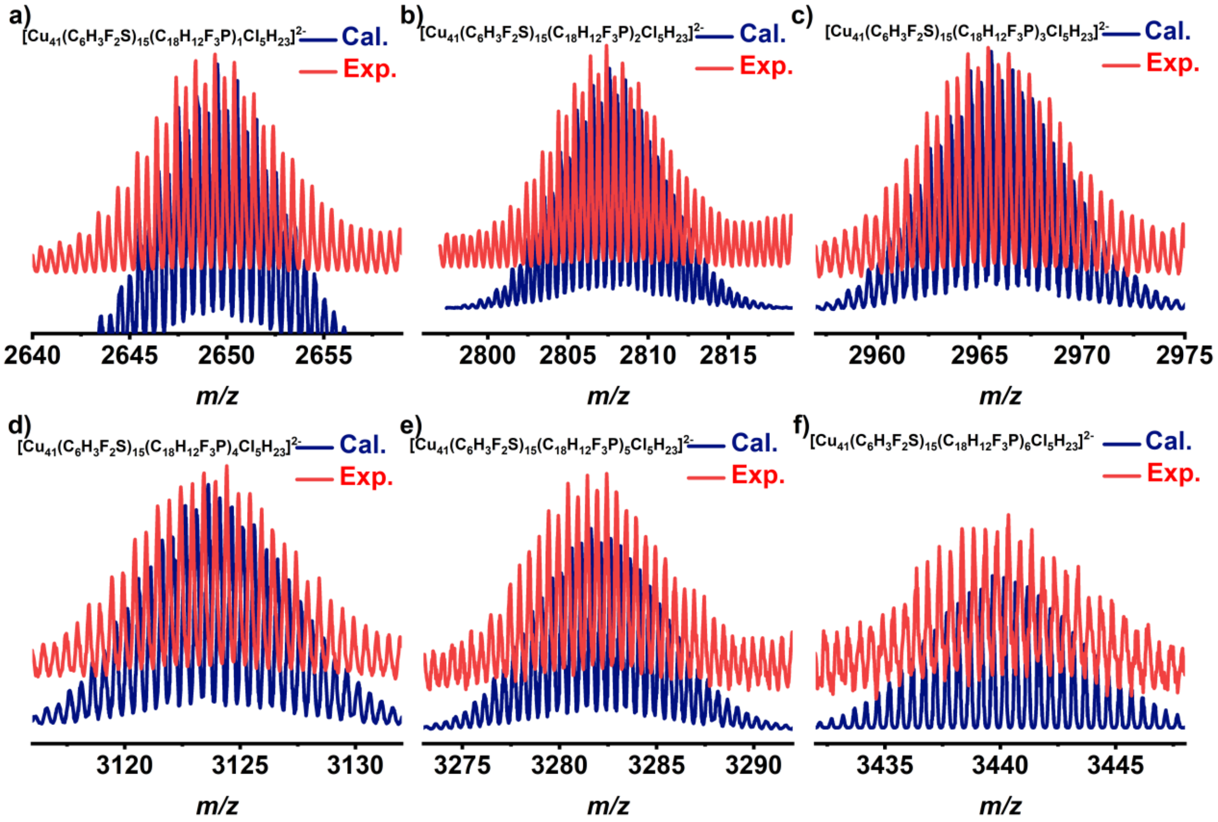

Figure S6. Comparison of the calculated (blue) and experimental (red) isotope distribution patterns of a) $[Cu_{41}(SC_6H_3F_2)_{15}Cl_3(P(PhF)_3)_1(H)_{25}]^{2-}$, b) $[Cu_{41}(SC_6H_3F_2)_{15}Cl_3(P(PhF)_3)_2(H)_{25}]^{2-}$, c) $[Cu_{41}(SC_6H_3F_2)_{15}Cl_3(P(PhF)_3)_3(H)_{25}]^{2-}$, d) $[Cu_{41}(SC_6H_3F_2)_{15}Cl_3(P(PhF)_3)_4(H)_{25}]^{2-}$, e) $[Cu_{41}(SC_6H_3F_2)_{15}Cl_3(P(PhF)_3)_5(H)_{25}]^{2-}$ and f) $[Cu_{41}(SC_6H_3F_2)_{15}Cl_3(P(PhF)_3)_6(H)_{25}]^{2-}$.

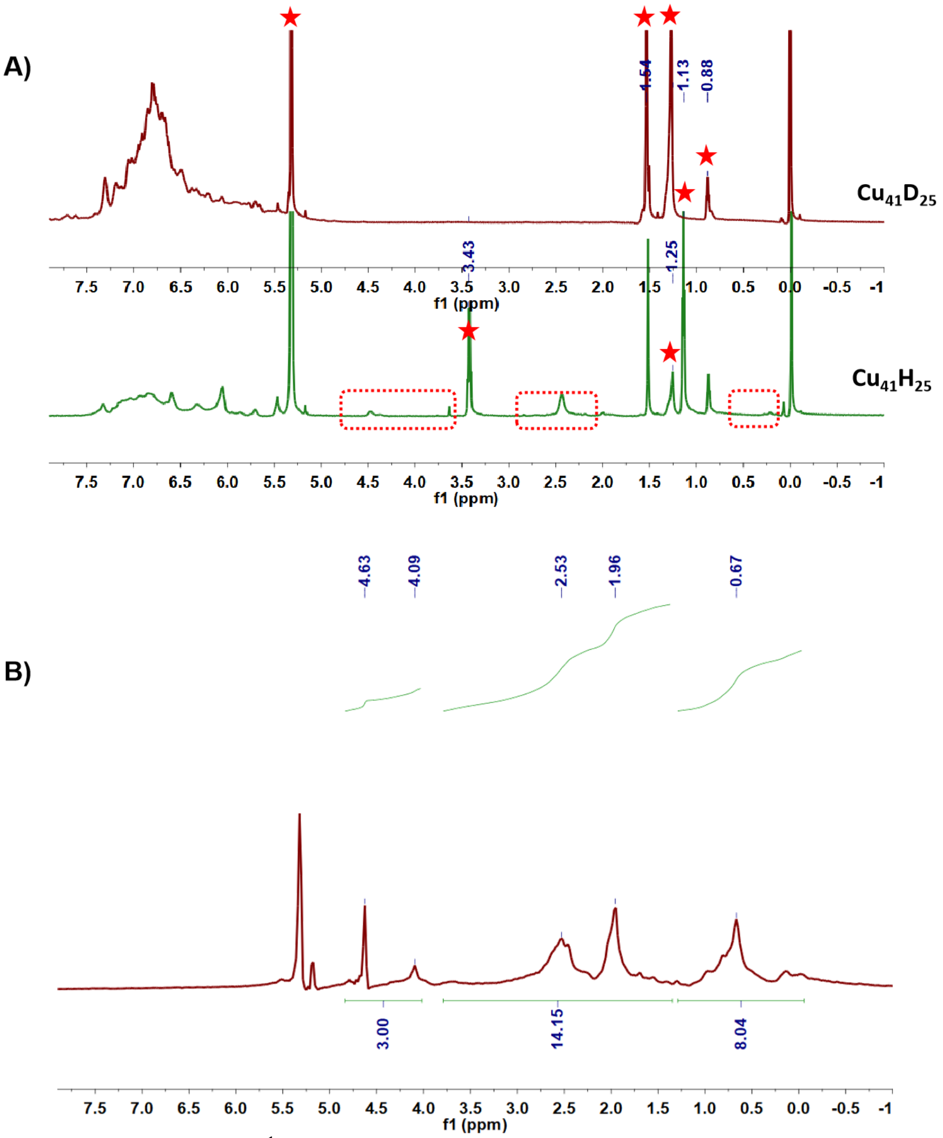

Figure S7. A) The ^{1}HNMR of $[Cu_{41}(SC_6H_3F_2)_{15}Cl_3(P(PhF)_3)_6(H)_{25}]^{2-}$ and $[Cu_{41}(SC_6H_3F_2)_{15}Cl_3(P(PhF)_3)_6(D)_{25}]^{2-}$ and B) The ^{2}HNMR of $[Cu_{41}(SC_6H_3F_2)_{15}Cl_3(P(PhF)_3)_6(D)_{25}]^{2-}$. The asterisk represents the signal peak of solvents.

	NMR Shift (ppm)
Atoms	Calc. (^1H)
H429, H438	2.0815
H430	4.8935
H431	7.7155
H432	6.0795
H433	3.5275
H434	4.3235
H435	3.6575
H436, H451	5.4735
H437	2.3895
H439	2.7835
H440	5.2725
H441	4.9855
H442	2.4585
H443	2.6135
H444, H447, H452	4.1315
H445	6.3115
H446	2.2415
H448	2.7005
H449	5.1345
H450	5.4015
H453	4.4935

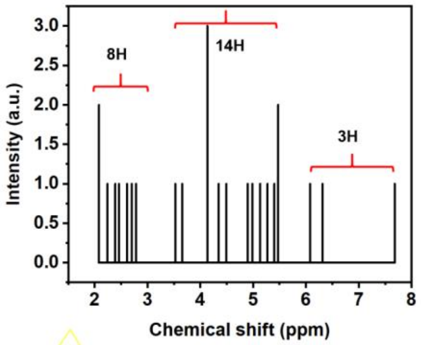

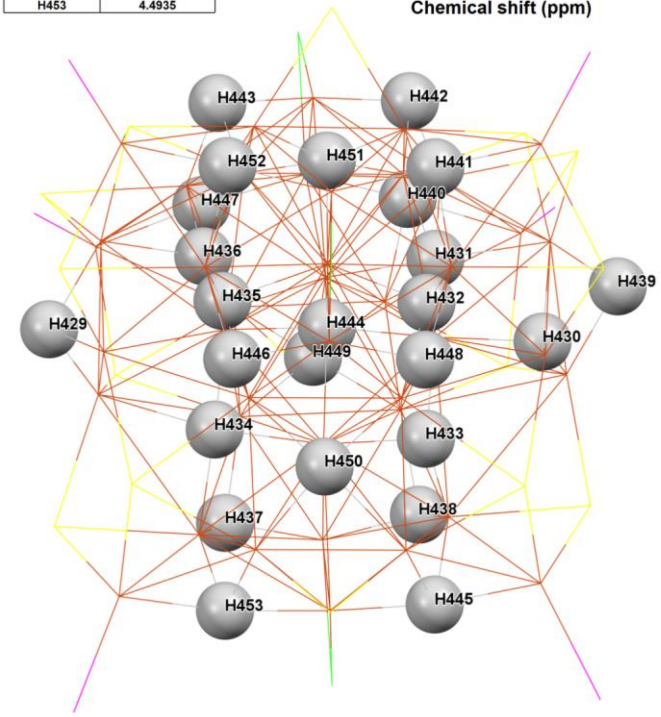

Figure S8. DFT calculations on the hydride chemical shifts of $[Cu_{41}(SC_6H_3F_2)_{15}Cl_3(P(PhF)_3)_6(H)_{25}]^{2-}$.

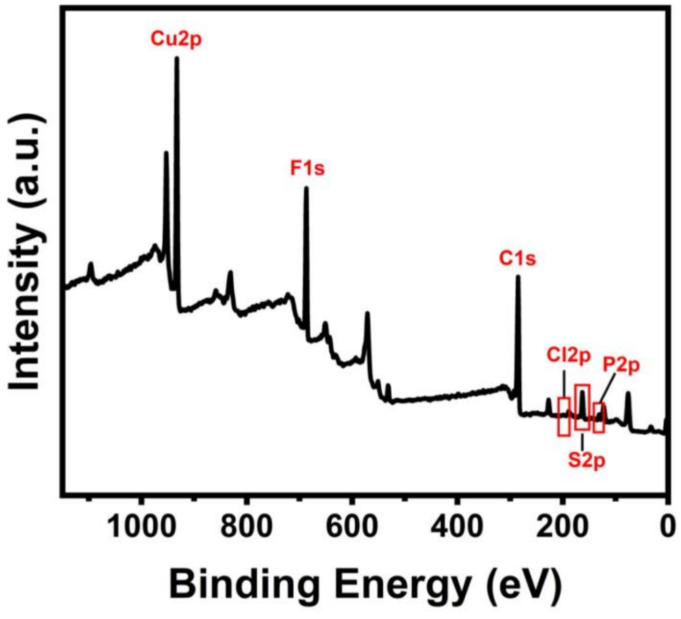

Figure S9. XPS spectra of $[Cu_{41}(SC_6H_3F_2)_{15}Cl_3(P(PhF)_3)_6(H)_{25}]^{2-}$.

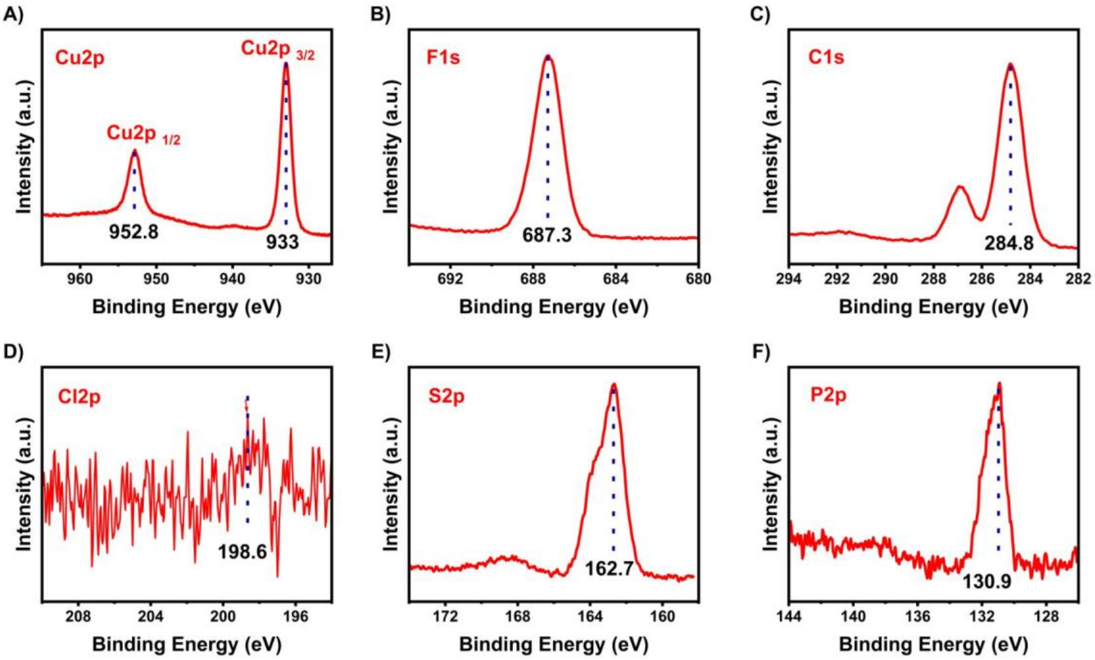

Figure S10. Survey spectrum of $[Cu_{41}(SC_6H_3F_2)_{15}Cl_3(P(PhF)_3)_6(H)_{25}]^{2-}$; (A-F) High-resolution XPS spectra of Cu2p、F1s、C1s、Cl2p、S2p and P2p.

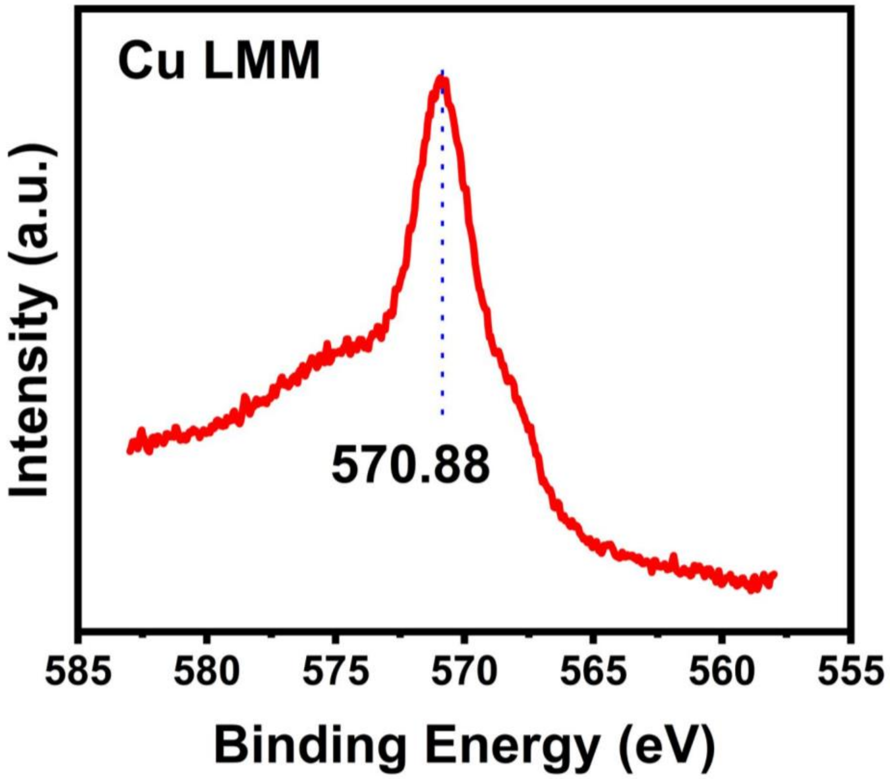

Figure S11. Cu LMM auger spectrum of $[Cu_{41}(SC_6H_3F_2)_{15}Cl_3(P(PhF)_3)_6(H)_{25}]^{2-}$.

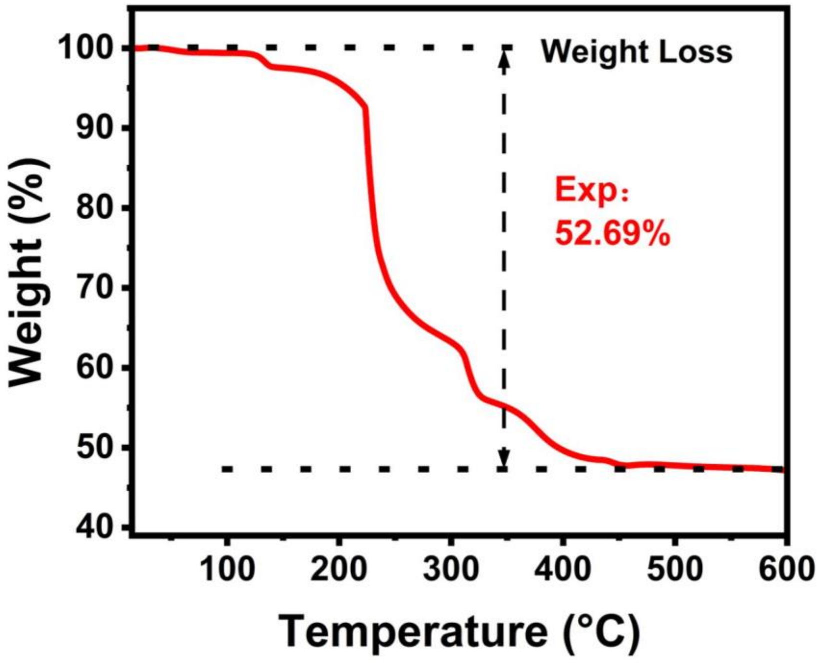

Figure S12. The thermogravimetric analysis (TGA) results of $[Cu_{41}(SC_6H_3F_2)_{15}Cl_3(P(PhF)_3)_6(H)_{25}]^{2}$-.

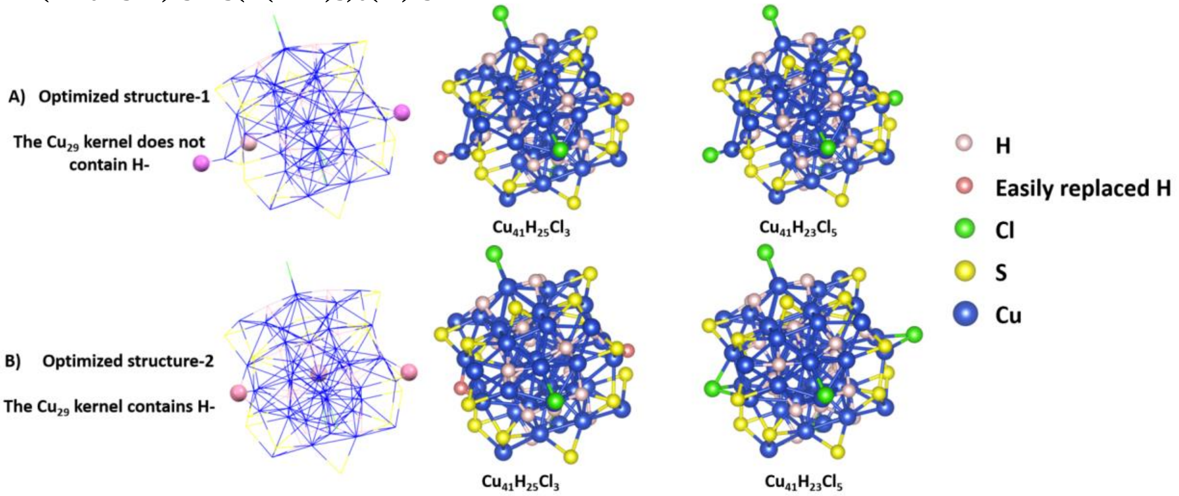

Figure S13. Optimized structures of $[Cu_{41}(SC_6H_3F_2)_{15}\mathbf{Cl_3}(P(PhF)_3)_6\mathbf{(H)_{25}}]^{2-}$, which the difference being whether the kernel has an H^- as well as the optimized structures of $[Cu_{41}(SC_6H_3F_2)_{15}\mathbf{Cl_5}(P(PhF)_3)_6\mathbf{(H)_{23}}]^{2-}$.

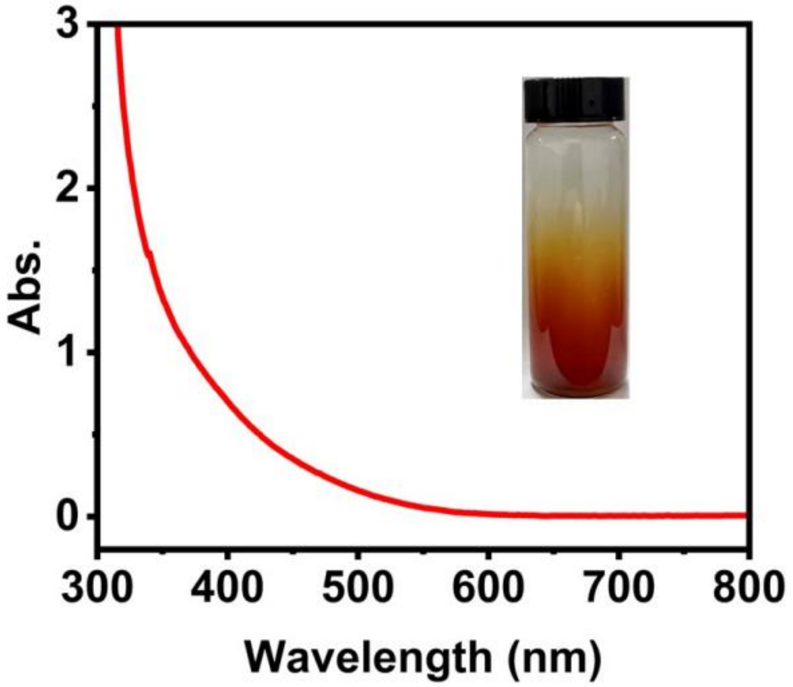

Figure S14. The optical absorption spectrum of $[Cu_{41}(SC_6H_3F_2)_{15}Cl_3(P(PhF)_3)_6(H)_{25}]^{2-}$ crystals dissolved in DCM. Inset: photograph of the DCM solution of $[Cu_{41}(SC_6H_3F_2)_{15}Cl_3(P(PhF)_3)_6(H)_{25}]^{2-}$.

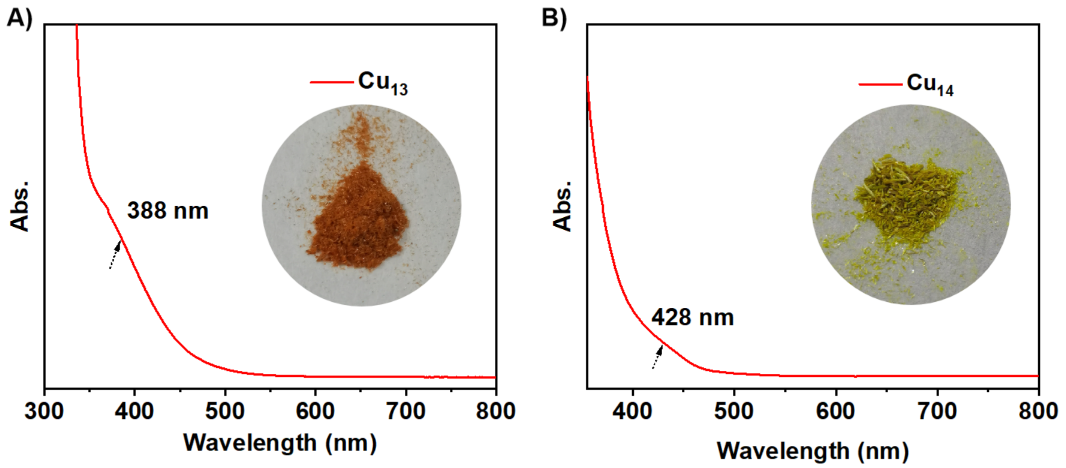

Figure S15. The UV-vis spectra of $[Cu_{13}(SC_6H_3F_2)_3(P(PhF)_3)_7H_{10}]^0$ (A) and $[Cu_{14}(SC_6H_3F_2)_3(PPh_3)_8H_{10}]^+$ (B). The insets show the crystals of the nanoclusters.

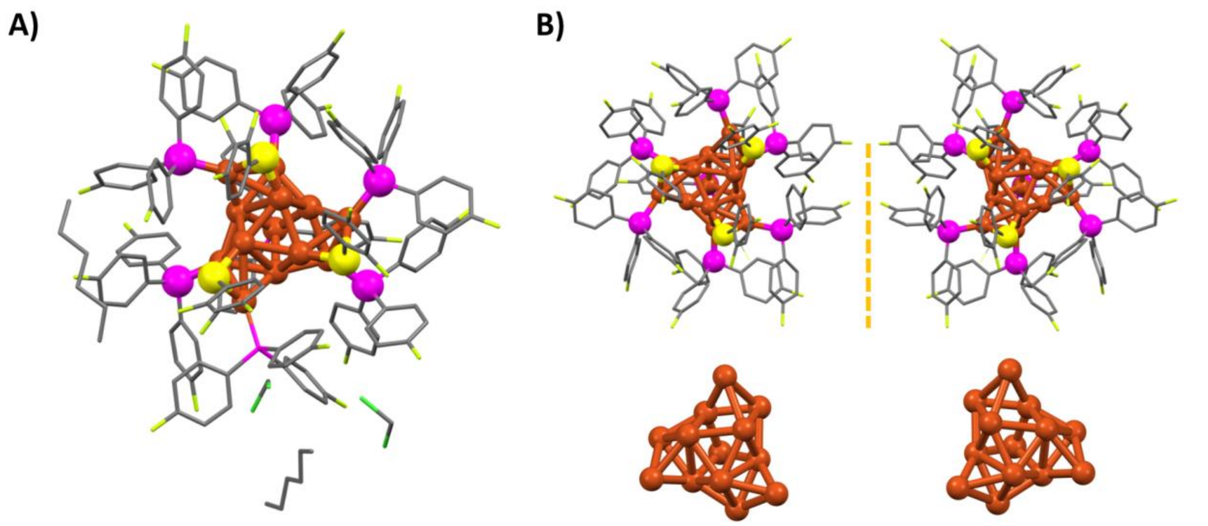

Figure S16. The overall structure of $[Cu_{13}(SC_6H_3F_2)_3(P(PhF)_3)_7H_{10}]^0$ (A) and enantiomers (B).

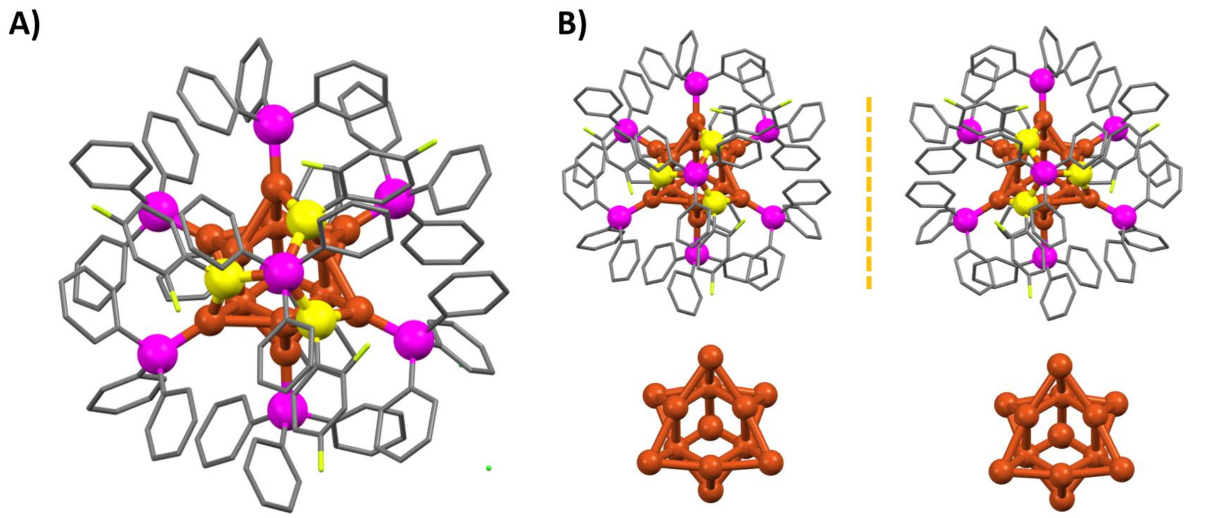

Figure S17. The overall structure of $[Cu_{14}(SC_6H_3F_2)_3(PPh_3)_8H_{10}]^+$ (A) and enantiomers (B).

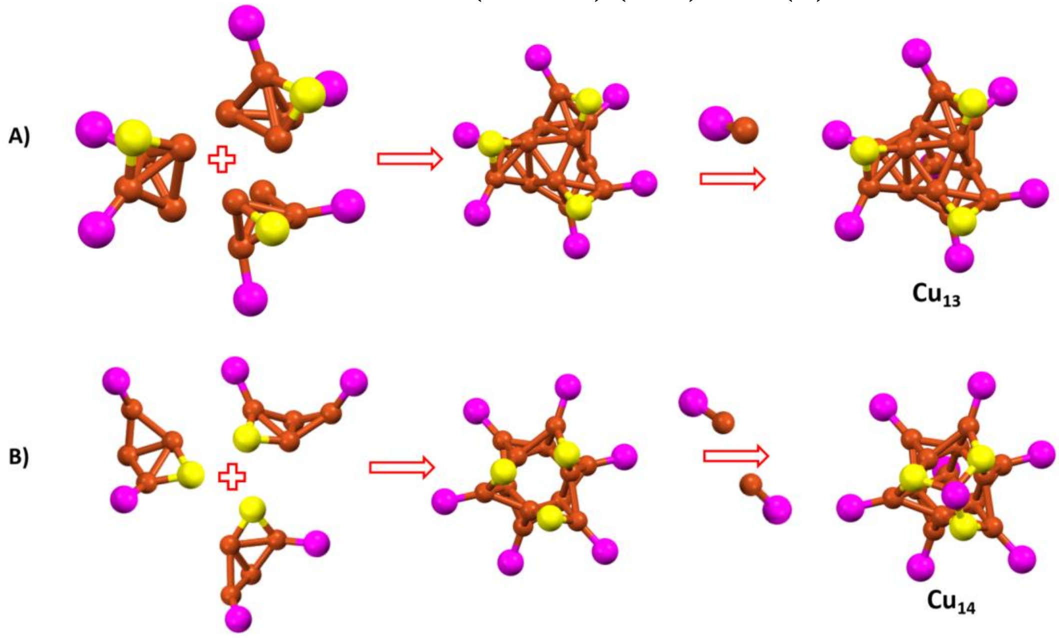

Figure S18. The structure splitting of $[Cu_{13}(SC_6H_3F_2)_3(P(PhF)_3)_7H_{10}]^0$ (A) and $[Cu_{14}(SC_6H_3F_2)_3(PPh_3)_8H_{10}]^+$ (B).

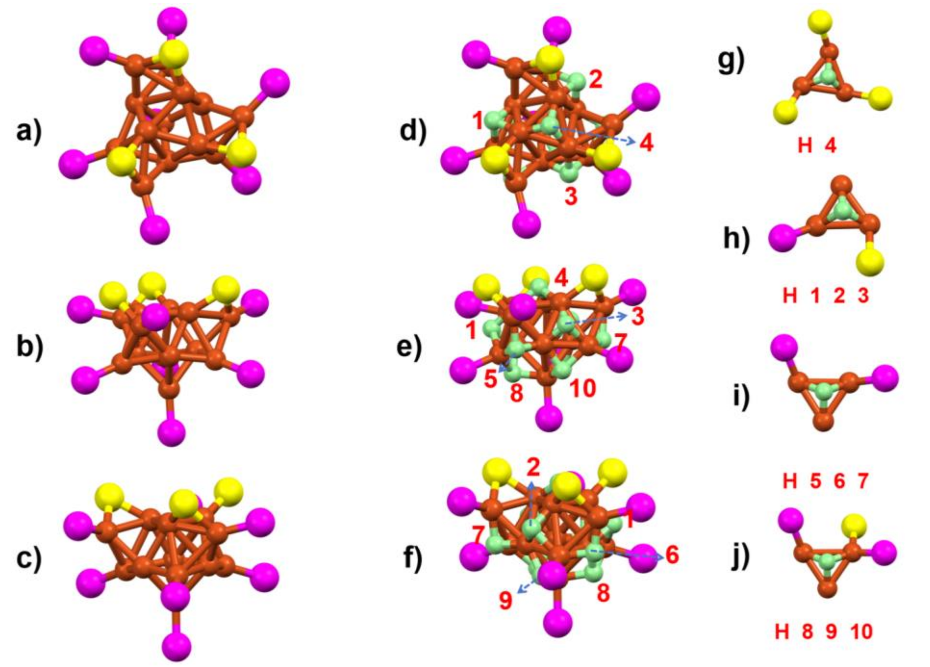

Figure S19. The top view (a), main view (b) and back view(c) of the $[Cu_{13}(SC_6H_3F_2)_3(P(PhF)_3)_7H_{10}]^0$ nanoclusters detetmined by X-ray crystallography without the the hydride.The top view (d), main view (e) and back view(f) of the optimized structure for the $[Cu_{13}(SC_6H_3F_2)_3(P(PhF)_3)_7H_{10}]^0$ nanoclusters with the hydride sites in the kernel obtained from DFT calculations. (g-j) The hydrides binding with Cu atoms can be observed to form four distinct groups based on the triangular Cu_3 coordination environment. Color code: brown, Cu; magenta, P; yellow, S; light green, μ_3-H in the kernel. The carbon terminals of the ligands are omitted for clarity.

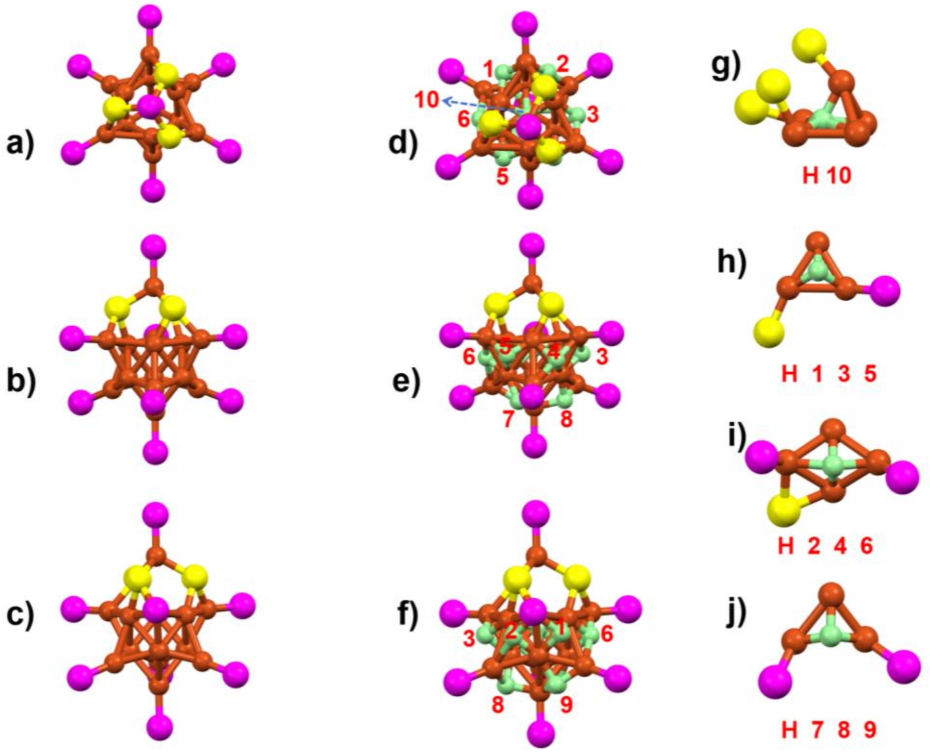

Figure S20. The top view (a), main view (b) and back view(c) of the $[Cu_{14}(SC_6H_3F_2)_3(PPh_3)_8H_{10}]^+$ nanoclusters detetmined by X-ray crystallography without the

the hydride. The top view (d), main view (e) and back view(f) of the optimized structure for the $[Cu_{14}(SC_6H_3F_2)_3(PPh_3)_8)_3H_{10}]^+$ nanoclusters with the hydride sites in the kernel obtained from DFT calculations. (g-j) The hydrides binding with Cu atoms can be observed to form four distinct groups based on the triangular $Cu_3/Cu_4/Cu_5$ coordination environment. Color code: brown, Cu; magenta, P; yellow, S; light green. The carbon terminals of the ligands are omitted for clarity.

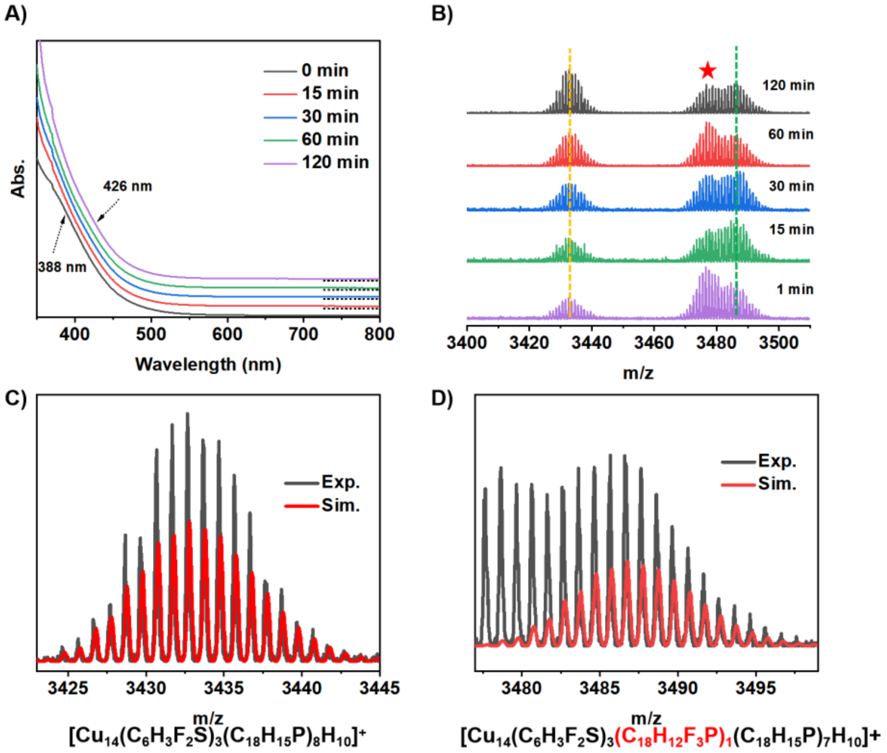

Figure S21. The transformation of $[Cu_{13}(SC_6H_3F_2)_3(P(PhF)_3)_7H_{10}]^0$ induced by addition of PPh_3 ligand. A) The variation trend of the UV-visible spectra of the $[Cu_{13}(SC_6H_3F_2)_3(P(PhF)_3)_7H_{10}]^0$ induced by addition of PPh_3 ligand; B) The variation trend of electrospray ionization mass spectrometry of samples. C) The calculated (red) and experimental (black) isotope distribution patterns of $[Cu_{14}(SC_6H_3F_2)_3(PPh_3)_8H_{10}]^+$. D) The calculated (red) and experimental (black) isotope distribution patterns of $[Cu_{14}(SC_6H_3F_2)_3(PPh_3)_7((P(PhF)_3)_1)H_{10}]^+$. The asterisk represents the signal peak of $[Cu_{14}(SC_6H_3F_2)_3(PPh_3)_8H_{10}+CH_3CH_2OH]^+$.

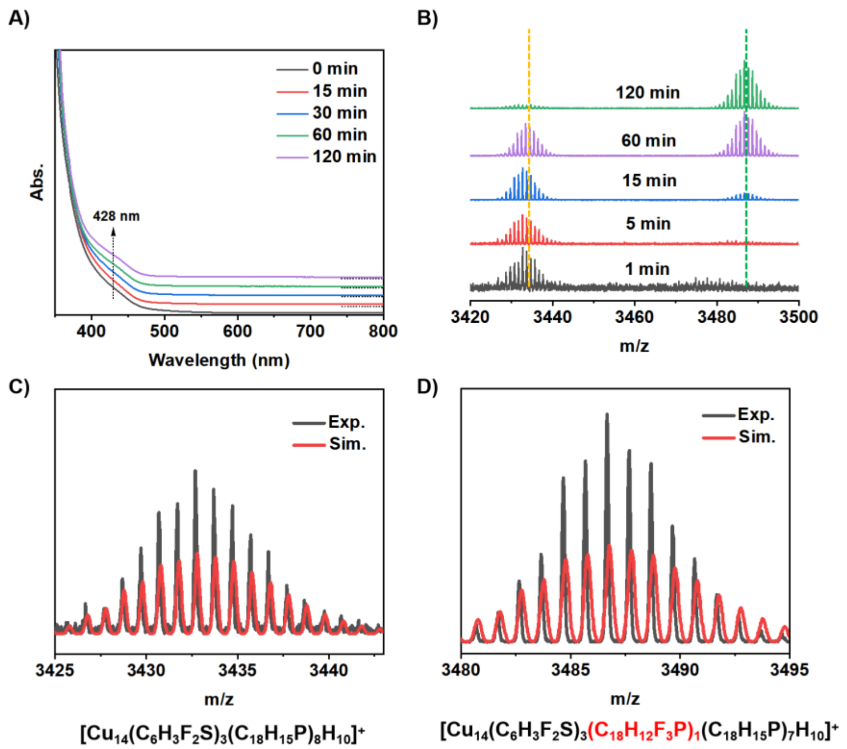

Figure S22. The transformation of $[Cu_{14}(SC_6H_3F_2)_3(PPh_3)_8H_{10}]^+$ induced by addition of $P(PhF)_3$ ligand. A) The variation trend of the UV-visible spectra of the $[Cu_{14}(SC_6H_3F_2)_3(PPh_3)_8H_{10}]^+$ induced by addition of $P(PhF)_3$ ligand; B) The variation trend of electrospray ionization mass spectrometry of samples. C) The calculated (red) and experimental (black) isotope distribution patterns of $[Cu_{14}(SC_6H_3F_2)_3(PPh_3)_8H_{10}]^+$. D) The calculated (red) and experimental (black) isotope distribution patterns of $[Cu_{14}(SC_6H_3F_2)_3(PPh_3)_7((P(PhF)_3)_1)H_{10}]^+$.

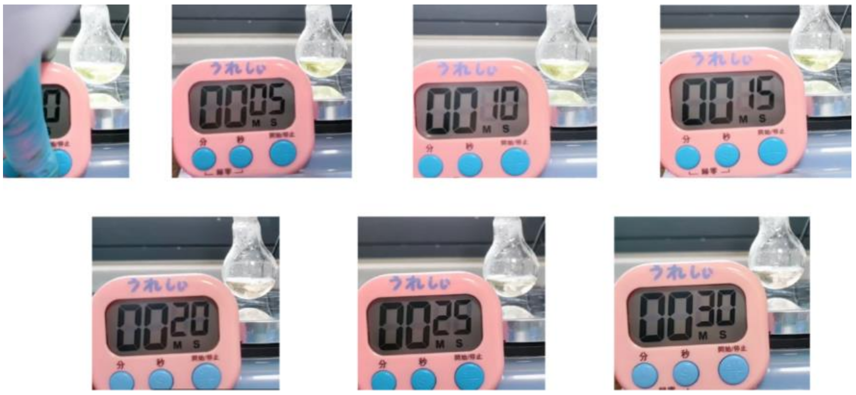

Figure S23. Digital photos of experimental phenomena of hydrogenation of p-nitrophenol (p-NP) to aminophenol (p-AP) with BH_4^- catalysis within 30s.

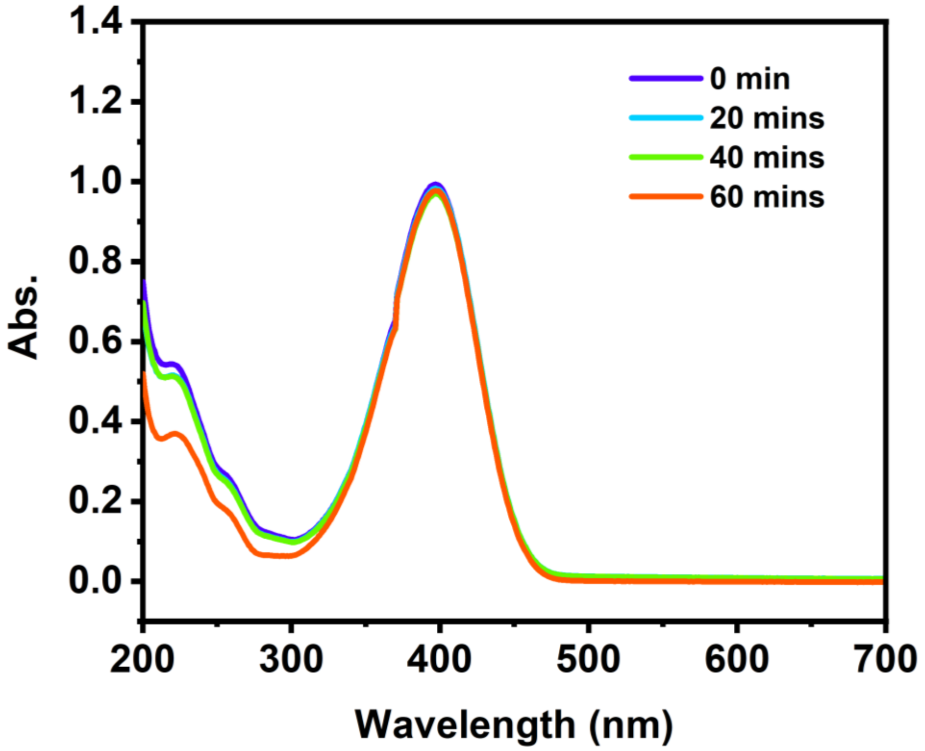

Figure S24. Time-dependent UV-visible absorption spectra of reduction of p-NP by $NaBH_4$ in the absence of $[Cu_{41}(SC_6H_3F_2)_{15}Cl_3(P(PhF)_3)_6(H)_{25}]^{2-}$ catalyst .

Table S1. Crystal data and structure refinement for Cu_{41}.

Identification code	Cu_{41}
Empirical formula	$C_{201}H_{123}Cl_9Cu_{41}F_{48}P_6S_{15}$
Formula weight	7040.90
Temperature/K	120
Crystal system	monoclinic
Space group	$P2_1/c$
a/Å	20.3129(15)
b/Å	33.065(2)
c/Å	36.168(2)
α/°	90
β/°	90.626(6)
γ/°	90
Volume/Å^3	24291(3)
Z	4
Radiation	CuKα (λ = 1.54186)
2Θ range for data collection/°	3.62 to 129.998
Index ranges	$-18 \le h \le 23$, $-28 \le k \le 38$, $-41 \le l \le 42$
Final R indexes [I>=2σ (I)]	R1 = 0.0636, wR2 = 0.1213
Final R indexes [all data]	R1 = 0.1029, wR2 = 0.1264
Largest diff. peak/hole / e Å^{-3}	0.61/-0.60

Table S2. Crystal data and structure refinement for Cu_{13}.

Identification code	Cu_{13}
Empirical formula	$C_{156.33}H_{121.67}Cl_{2.67}Cu_{13}F_{27}P_7S_3$
Formula weight	3746.74
Temperature/K	120
Crystal system	Monoclinic
Space group	$P2_1/n$
a/Å	26.6635(9)
b/Å	22.3694(10)
c/Å	28.8686(14)
α/°	90
β/°	109.177(3)
γ/°	90
Volume/$Å^3$	16263.1(12)
Z	4
Radiation	CuKα (λ = 1.54186)
2Θ range for data collection/°	8.65 to 139.662
Index ranges	$-32 \leq h \leq 22$, $-25 \leq k \leq 27$, $-35 \leq l \leq 30$
Final R indexes [I>=2σ (I)]	R_1 = 0.0620, wR_2 = 0.1715
Final R indexes [all data]	R_1 = 0.0761, wR_2 = 0.1854
Largest diff. peak/hole / e $Å^{-3}$	1.60/-1.35

Table S3. Crystal data and structure refinement for Cu_{14}.

Identification code	Cu_{14}
Empirical formula	$C_{163}H_{129}Cu_{14}F_6O_3P_8S_3$
Formula weight	3484.82
Temperature/K	120
Crystal system	trigonal
Space group	R-3c
a/Å	17.397(3)
b/Å	17.397(3)
c/Å	179.53(2)
α/°	90
β/°	90
γ/°	120
Volume/$Å^3$	47054(16)
Z	12
Radiation	CuKα (λ = 1.54186)
2Θ range for data collection/°	7.068 to 139.246
Index ranges	$-19 \leq h \leq 21$, $-21 \leq k \leq 18$, $-100 \leq l \leq 216$
Final R indexes [I>=2σ (I)]	R_1 = 0.0654, wR_2 = 0.1821
Final R indexes [all data]	R_1 = 0.0903, wR_2 = 0.2066
Largest diff. peak/hole / e $Å^{-3}$	1.42/-0.56
