# Supplementary material for: Total Structure, Structural Transformation and Catalytic Hydrogenation of [Cu41(SC6H3F2)15Cl3(P(PhF)3)6(H)25]2− Constructed from Twisted Cu13 Units
Source: Adv Sci (Weinh). 2023 Dec 8;11(7):2307085. doi: 10.1002/advs.202307085 (PMC10870033; doi:10.1002/advs.202307085)

checkCIF/PLATON report

You have not supplied any structure factors. As a result the full set of tests cannot be run.

THIS REPORT IS FOR GUIDANCE ONLY. IF USED AS PART OF A REVIEW PROCEDURE FOR PUBLICATION, IT SHOULD NOT REPLACE THE EXPERTISE OF AN EXPERIENCED CRYSTALLOGRAPHIC REFEREE.

No syntax errors found. CIF dictionary Interpreting this report

Datablock: 1_sq

Bond precision: C-C = 0.0083 A Wavelength=1.54186

Cell:	a=26.6635(9)	b=22.3694(10)	c=28.8686(14)
	alpha=90	beta=109.177(3)	gamma=90
Temperature:	120 K		

	Calculated	Reported
Volume	16263.1(12)	16263.1(12)
Space group	P 21/n	P 1 21/n 1
Hall group	-P 2yn	-P 2yn
Moiety formula	3(C24 H15 Cu2 F5 P S), 3(C18 H12 Cu2 F3 P), C18 H12 Cu F3 P, 2(	C144 H93 Cu13 F27 P7 S3, 1.33(C H2 Cl2), 2(C6 H14)
Sum formula	C157.33 H123.67 Cl2.67 Cu13 F27 P7 S3 [+ solvent]	C156.33 H121.67 Cl2.67 Cu13 F27 P7 S3
Mr	3760.88	3746.74
Dx,g cm-3	1.536	1.530
Z	4	4
Mu (mm-1)	3.874	3.871
F000	7544.0	7512.0
F000'	7476.19	
h,k,lmax	32,27,35	32,27,35
Nref	30763	30054
Tmin,Tmax		
Tmin'		

Correction method= Not given

Data completeness= 0.977 Theta(max)= 69.831

R(reflections)= 0.0620(24100)   wR2(reflections)= 0.1854(30054)

S = 1.035   Npar= 1912

The following ALERTS were generated. Each ALERT has the format
test-name_ALERT_alert-type_alert-level.
Click on the hyperlinks for more details of the test.

Alert level A

```
PLAT308_ALERT_2_A Single Bonded Metal Atom in Structure  (Unusual)       Cu04 Check
PLAT308_ALERT_2_A Single Bonded Metal Atom in Structure  (Unusual)       Cu0A Check
PLAT308_ALERT_2_A Single Bonded Metal Atom in Structure  (Unusual)       Cu05 Check
PLAT308_ALERT_2_A Single Bonded Metal Atom in Structure  (Unusual)       Cu08 Check
PLAT308_ALERT_2_A Single Bonded Metal Atom in Structure  (Unusual)       Cu0D Check
PLAT308_ALERT_2_A Single Bonded Metal Atom in Structure  (Unusual)       Cu02 Check
PLAT308_ALERT_2_A Single Bonded Metal Atom in Structure  (Unusual)       Cu07 Check
```

Alert level C

```
PLAT052_ALERT_1_C Info on Absorption Correction Method   Not Given     Please Do !
PLAT053_ALERT_1_C Minimum Crystal Dimension Missing (or Error) ...     Please Check
PLAT054_ALERT_1_C Medium  Crystal Dimension Missing (or Error) ...     Please Check
PLAT055_ALERT_1_C Maximum Crystal Dimension Missing (or Error) ...     Please Check
PLAT077_ALERT_4_C Unitcell Contains Non-integer Number of Atoms ..     Please Check
PLAT213_ALERT_2_C Atom F01D           has ADP max/min Ratio .....         3.5 prolat
PLAT213_ALERT_2_C Atom F02G           has ADP max/min Ratio .....         3.6 prolat
PLAT242_ALERT_2_C Low    'MainMol' Ueq as Compared to Neighbors of       C05H Check
PLAT244_ALERT_4_C Low    'Solvent' Ueq as Compared to Neighbors of       C0AA Check
PLAT260_ALERT_2_C Large Average Ueq of Residue Including        C1      0.124 Check
PLAT260_ALERT_2_C Large Average Ueq of Residue Including        C6      0.220 Check
PLAT341_ALERT_3_C Low Bond Precision on  C-C Bonds ..............    0.00832 Ang.
```

Alert level G

```
FORMU01_ALERT_1_G  There is a discrepancy between the atom counts in the
            _chemical_formula_sum and _chemical_formula_moiety. This is
            usually due to the moiety formula being in the wrong format.
            Atom count from _chemical_formula_sum:   C156.33 H121.67 Cl2.67 Cu13 F
            Atom count from _chemical_formula_moiety:C157.3300 H123.66 Cl2.66 Cu13
FORMU01_ALERT_2_G  There is a discrepancy between the atom counts in the
            _chemical_formula_sum and the formula from the _atom_site* data.
            Atom count from _chemical_formula_sum:C156.33 H121.67 Cl2.67 Cu13 F27
            Atom count from the _atom_site data:  C157.3333 H123.6665 Cl2.666600 C
CELLZ01_ALERT_1_G Difference between formula and atom_site contents detected.
CELLZ01_ALERT_1_G ALERT: Large difference may be due to a
            symmetry error - see SYMMG tests
           From the CIF: _cell_formula_units_Z    4
           From the CIF: _chemical_formula_sum  C156.33 H121.67 Cl2.67 Cu13 F27 P7
           TEST: Compare cell contents of formula and atom_site data

           atom    Z*formula  cif sites diff
           C        625.32    629.33   -4.01
           H        486.68    494.67   -7.99
           Cl        10.68     10.67    0.01
```

```
          Cu        52.00     52.00    0.00
          F        108.00    108.00    0.00
          P         28.00     28.00    0.00
          S         12.00     12.00    0.00
PLAT002_ALERT_2_G Number of Distance or Angle Restraints on AtSite         12 Note
PLAT003_ALERT_2_G Number of Uiso or Uij Restrained non-H Atoms ...       158 Report
PLAT041_ALERT_1_G Calc. and Reported SumFormula    Strings  Differ     Please Check
PLAT042_ALERT_1_G Calc. and Reported MoietyFormula Strings  Differ     Please Check
PLAT072_ALERT_2_G SHELXL First  Parameter in WGHT  Unusually Large       0.14 Report
PLAT083_ALERT_2_G SHELXL Second Parameter in WGHT  Unusually Large       6.73 Why ?
PLAT172_ALERT_4_G The CIF-Embedded .res File Contains DFIX Records         11 Report
PLAT178_ALERT_4_G The CIF-Embedded .res File Contains SIMU Records          1 Report
PLAT186_ALERT_4_G The CIF-Embedded .res File Contains ISOR Records          1 Report
PLAT188_ALERT_3_G A Non-default SIMU Restraint Value has been used     0.0100 Report
PLAT300_ALERT_4_G Atom Site Occupancy of Cl1A       Constrained at     0.3333 Check
PLAT300_ALERT_4_G Atom Site Occupancy of Cl0A       Constrained at     0.3333 Check
PLAT300_ALERT_4_G Atom Site Occupancy of C2         Constrained at     0.3333 Check
PLAT300_ALERT_4_G Atom Site Occupancy of H2A        Constrained at     0.3333 Check
PLAT300_ALERT_4_G Atom Site Occupancy of H2B        Constrained at     0.3333 Check
PLAT302_ALERT_4_G Anion/Solvent/Minor-Residue Disorder (Resd 11  )       100% Note
PLAT304_ALERT_4_G Non-Integer Number of Atoms in ..... (Resd 11  )       1.67 Check
PLAT434_ALERT_2_G Short Inter HL..HL Contact F00T     ..F016     .       2.78 Ang.
                                              1-x,1-y,1-z  =      3_666 Check
PLAT434_ALERT_2_G Short Inter HL..HL Contact F012     ..F018     .       2.75 Ang.
                                        1/2+x,1/2-y,1/2+z  =      4_666 Check
PLAT606_ALERT_4_G Solvent Accessible VOID(S) in Structure ........          ! Info
PLAT720_ALERT_4_G Number of Unusual/Non-Standard Labels ..........        295 Note
PLAT764_ALERT_4_G Overcomplete CIF Bond List Detected (Rep/Expd) .       1.12 Ratio
PLAT794_ALERT_5_G Tentative Bond Valency for Cu01      (I)       .       0.62 Info
PLAT794_ALERT_5_G Tentative Bond Valency for Cu04      (I)       .       0.37 Info
PLAT794_ALERT_5_G Tentative Bond Valency for Cu05      (I)       .       0.38 Info
PLAT794_ALERT_5_G Tentative Bond Valency for Cu07      (I)       .       0.28 Info
PLAT794_ALERT_5_G Tentative Bond Valency for Cu0A      (I)       .       0.36 Info
PLAT794_ALERT_5_G Tentative Bond Valency for Cu0B      (I)       .       0.62 Info
PLAT794_ALERT_5_G Tentative Bond Valency for Cu0C      (I)       .       0.63 Info
PLAT860_ALERT_3_G Number of Least-Squares Restraints .............       4278 Note
PLAT869_ALERT_4_G ALERTS Related to the Use of SQUEEZE  Suppressed          ! Info
PLAT883_ALERT_1_G No Info/Value for _atom_sites_solution_primary .     Please Do !
PLAT933_ALERT_2_G Number of HKL-OMIT Records in Embedded .res File         13 Note
PLAT941_ALERT_3_G Average HKL Measurement Multiplicity ...........        4.3 Low
```

---

   7 **ALERT level A** = Most likely a serious problem - resolve or explain
   0 **ALERT level B** = A potentially serious problem, consider carefully
  12 **ALERT level C** = Check. Ensure it is not caused by an omission or oversight
  38 **ALERT level G** = General information/check it is not something unexpected

  10 ALERT type 1 CIF construction/syntax error, inconsistent or missing data
  20 ALERT type 2 Indicator that the structure model may be wrong or deficient
   4 ALERT type 3 Indicator that the structure quality may be low
  16 ALERT type 4 Improvement, methodology, query or suggestion
   7 ALERT type 5 Informative message, check

It is advisable to attempt to resolve as many as possible of the alerts in all categories. Often the minor alerts point to easily fixed oversights, errors and omissions in your CIF or refinement strategy, so attention to these fine details can be worthwhile. In order to resolve some of the more serious problems it may be necessary to carry out additional measurements or structure refinements. However, the purpose of your study may justify the reported deviations and the more serious of these should normally be commented upon in the discussion or experimental section of a paper or in the "special_details" fields of the CIF. checkCIF was carefully designed to identify outliers and unusual parameters, but every test has its limitations and alerts that are not important in a particular case may appear. Conversely, the absence of alerts does not guarantee there are no aspects of the results needing attention. It is up to the individual to critically assess their own results and, if necessary, seek expert advice.

Publication of your CIF in IUCr journals

A basic structural check has been run on your CIF. These basic checks will be run on all CIFs submitted for publication in IUCr journals (*Acta Crystallographica*, *Journal of Applied Crystallography*, *Journal of Synchrotron Radiation*); however, if you intend to submit to *Acta Crystallographica Section C* or *E* or *IUCrData*, you should make sure that full publication checks are run on the final version of your CIF prior to submission.

Publication of your CIF in other journals

Please refer to the *Notes for Authors* of the relevant journal for any special instructions relating to CIF submission.

PLATON version of 06/07/2023; check.def file version of 30/06/2023

Datablock 1_sq - ellipsoid plot

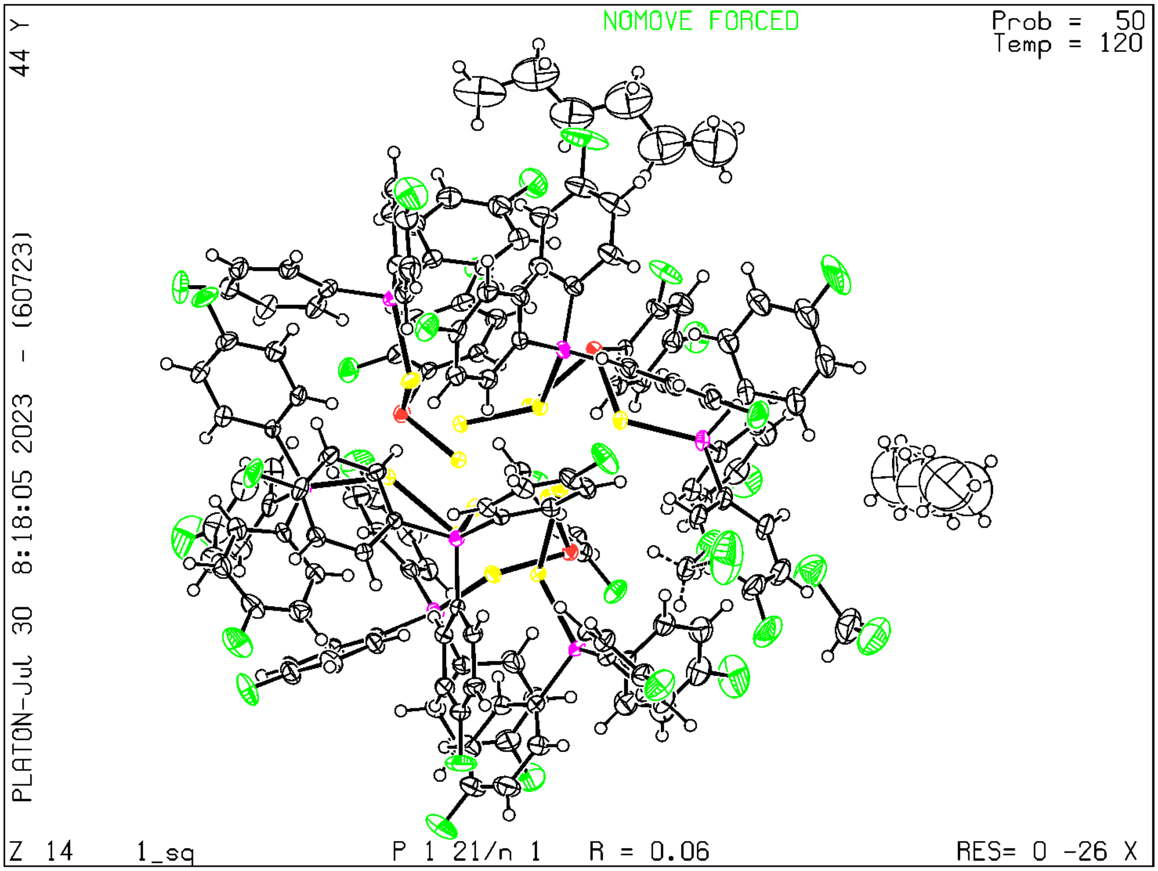

Supplement: Supplementary file 2 — Supporting Information [file ADVS-11-2307085-s002.zip › advs202307085-sup-0002-cif/checkcif-Cu13.pdf]
