# Supplementary material for: Total Structure, Structural Transformation and Catalytic Hydrogenation of [Cu41(SC6H3F2)15Cl3(P(PhF)3)6(H)25]2− Constructed from Twisted Cu13 Units
Source: Adv Sci (Weinh). 2023 Dec 8;11(7):2307085. doi: 10.1002/advs.202307085 (PMC10870033; doi:10.1002/advs.202307085)

checkCIF/PLATON report

You have not supplied any structure factors. As a result the full set of tests cannot be run.

THIS REPORT IS FOR GUIDANCE ONLY. IF USED AS PART OF A REVIEW PROCEDURE FOR PUBLICATION, IT SHOULD NOT REPLACE THE EXPERTISE OF AN EXPERIENCED CRYSTALLOGRAPHIC REFEREE.

No syntax errors found. CIF dictionary Interpreting this report

Datablock: op

```
Bond precision:   C-C = 0.0128 A          Wavelength=1.54186

Cell:               a=17.397(3)      b=17.397(3)      c=179.53(2)
                    alpha=90         beta=90          gamma=120
Temperature:        120 K
```

	Calculated	Reported
Volume	47056(17)	47054(16)
Space group	R -3 c	R -3 c
Hall group	-R 3 2"c	-R 3 2"c
Moiety formula	C90 H69 Cu7 F6 P4 S3, 3(C18 H15 Cu2 P), C18 H15 Cu P, C O3 [+ s	1(C162 H129 Cu14 F6 P8 S3), 1(C1 O3)
Sum formula	C163 H129 Cu14 F6 O3 P8 S3 [+ solvent]	C162 H129 Cl0.08 Cu14 F6 N1.22 O2.60 P8 S3
Mr	3483.30	3484.82
Dx,g cm-3	1.475	1.476
Z	12	12
Mu (mm-1)	3.600	3.613
F000	21108.0	21117.0
F000’	20857.37	
h,k,lmax	21,21,217	21,21,216
Nref	9867	9695
Tmin,Tmax		
Tmin’		

```
Correction method= Not given

Data completeness= 0.983            Theta(max)= 69.623
```

R(reflections)= 0.0654(6912) wR2(reflections)= 0.2066(9695)

S = 1.031 Npar= 592

The following ALERTS were generated. Each ALERT has the format
test-name_ALERT_alert-type_alert-level.
Click on the hyperlinks for more details of the test.

Alert level A

PLAT308_ALERT_2_A Single Bonded Metal Atom in Structure (Unusual) Cu04 Check
PLAT308_ALERT_2_A Single Bonded Metal Atom in Structure (Unusual) Cu02 Check
PLAT308_ALERT_2_A Single Bonded Metal Atom in Structure (Unusual) Cu01 Check

---

Alert level C

PLAT052_ALERT_1_C Info on Absorption Correction Method Not Given Please Do !
PLAT053_ALERT_1_C Minimum Crystal Dimension Missing (or Error) ... Please Check
PLAT054_ALERT_1_C Medium Crystal Dimension Missing (or Error) ... Please Check
PLAT055_ALERT_1_C Maximum Crystal Dimension Missing (or Error) ... Please Check
PLAT094_ALERT_2_C Ratio of Maximum / Minimum Residual Density .... 2.51 Report
PLAT234_ALERT_4_C Large Hirshfeld Difference C3AA --C0BA . 0.21 Ang.
PLAT241_ALERT_2_C High 'MainMol' Ueq as Compared to Neighbors of C53 Check
PLAT260_ALERT_2_C Large Average Ueq of Residue Including O1 0.289 Check
PLAT309_ALERT_2_C Single Bonded Oxygen (C-O > 1.3 Ang) .... O1 Check
PLAT331_ALERT_2_C Small Aver Phenyl C-C Dist C0AA --C19 . 1.37 Ang.
PLAT334_ALERT_2_C Small <C-C> Benzene Dist. C2 -C9BA . 1.37 Ang.
PLAT341_ALERT_3_C Low Bond Precision on C-C Bonds .... 0.01283 Ang.

Alert level G

FORMU01_ALERT_1_G There is a discrepancy between the atom counts in the
_chemical_formula_sum and _chemical_formula_moiety. This is
usually due to the moiety formula being in the wrong format.
Atom count from _chemical_formula_sum: C162 H129 Cl.08 Cu14 F6 N1.22
Atom count from _chemical_formula_moiety:C163 H129 Cu14 F6 O3 P8 S3
FORMU01_ALERT_2_G There is a discrepancy between the atom counts in the
_chemical_formula_sum and the formula from the _atom_site* data.
Atom count from _chemical_formula_sum:C162 H129 Cl.08 Cu14 F6 N1.22 O2
Atom count from the _atom_site data: C163.0016 H129.0012 Cu14. F6. O3
CELLZ01_ALERT_1_G Difference between formula and atom_site contents detected.
CELLZ01_ALERT_1_G ALERT: Large difference may be due to a
symmetry error - see SYMMG tests
From the CIF: _cell_formula_units_Z 12
From the CIF: _chemical_formula_sum C162 H129 Cl0.08 Cu14 F6 N1.22 O2.
TEST: Compare cell contents of formula and atom_site data

atom	Z*formula	cif sites	diff
C	1943.98	1956.00	-12.02
H	1547.98	1548.00	-0.02
Cl	0.96	0.00	0.96
Cu	168.00	168.00	-0.00
F	72.00	72.00	-0.00
N	14.64	0.00	14.64
O	31.20	36.00	-4.80

```
          P         96.00     96.00   -0.00
          S         36.00     36.00   -0.00
PLAT003_ALERT_2_G Number of Uiso or Uij Restrained non-H Atoms ...          2 Report
PLAT041_ALERT_1_G Calc. and Reported SumFormula    Strings  Differ     Please Check
PLAT042_ALERT_1_G Calc. and Reported MoietyFormula Strings  Differ     Please Check
PLAT072_ALERT_2_G SHELXL First  Parameter in WGHT  Unusually Large       0.12 Report
PLAT083_ALERT_2_G SHELXL Second Parameter in WGHT  Unusually Large     258.48 Why ?
PLAT100_ALERT_5_G A Non-Integer Z value Reported in the CIF ......     12.000 Check
PLAT177_ALERT_4_G The CIF-Embedded .res File Contains DELU Records          1 Report
PLAT186_ALERT_4_G The CIF-Embedded .res File Contains ISOR Records          1 Report
PLAT192_ALERT_3_G A Non-default DELU Restraint Value for First Par     0.0010 Report
PLAT192_ALERT_3_G A Non-default DELU Restraint Value for SecondPar     0.0010 Report
PLAT344_ALERT_2_G Unusual sp?       Angle Range in Solvent/Ion for         C1 Check
PLAT605_ALERT_4_G Largest Solvent Accessible VOID in the Structure        372 A**3
PLAT720_ALERT_4_G Number of Unusual/Non-Standard Labels ..........         49 Note
PLAT764_ALERT_4_G Overcomplete CIF Bond List Detected (Rep/Expd) .       1.21 Ratio
PLAT794_ALERT_5_G Tentative Bond Valency for Cu01      (I)       .       0.27 Info
PLAT794_ALERT_5_G Tentative Bond Valency for Cu04      (I)       .       0.37 Info
PLAT794_ALERT_5_G Tentative Bond Valency for Cu05      (I)       .       0.63 Info
PLAT860_ALERT_3_G Number of Least-Squares Restraints .............          7 Note
PLAT868_ALERT_4_G ALERTS Due to the Use of _smtbx_masks Suppressed          ! Info
PLAT941_ALERT_3_G Average HKL Measurement Multiplicity ...........        4.6 Low
```

---

   3 **ALERT level A** = Most likely a serious problem - resolve or explain
   0 **ALERT level B** = A potentially serious problem, consider carefully
  12 **ALERT level C** = Check. Ensure it is not caused by an omission or oversight
  24 **ALERT level G** = General information/check it is not something unexpected

```
   9 ALERT type 1 CIF construction/syntax error, inconsistent or missing data
  14 ALERT type 2 Indicator that the structure model may be wrong or deficient
   5 ALERT type 3 Indicator that the structure quality may be low
   7 ALERT type 4 Improvement, methodology, query or suggestion
   4 ALERT type 5 Informative message, check
```

It is advisable to attempt to resolve as many as possible of the alerts in all categories. Often the minor alerts point to easily fixed oversights, errors and omissions in your CIF or refinement strategy, so attention to these fine details can be worthwhile. In order to resolve some of the more serious problems it may be necessary to carry out additional measurements or structure refinements. However, the purpose of your study may justify the reported deviations and the more serious of these should normally be commented upon in the discussion or experimental section of a paper or in the "special_details" fields of the CIF. checkCIF was carefully designed to identify outliers and unusual parameters, but every test has its limitations and alerts that are not important in a particular case may appear. Conversely, the absence of alerts does not guarantee there are no aspects of the results needing attention. It is up to the individual to critically assess their own results and, if necessary, seek expert advice.

Publication of your CIF in IUCr journals

A basic structural check has been run on your CIF. These basic checks will be run on all CIFs submitted for publication in IUCr journals (*Acta Crystallographica*, *Journal of Applied Crystallography*, *Journal of Synchrotron Radiation*); however, if you intend to submit to *Acta Crystallographica Section C* or *E* or *IUCrData*, you should make sure that full publication checks are run on the final version of your CIF prior to submission.

Publication of your CIF in other journals

Please refer to the *Notes for Authors* of the relevant journal for any special instructions relating to CIF submission.

PLATON version of 06/07/2023; check.def file version of 30/06/2023

Datablock op - ellipsoid plot

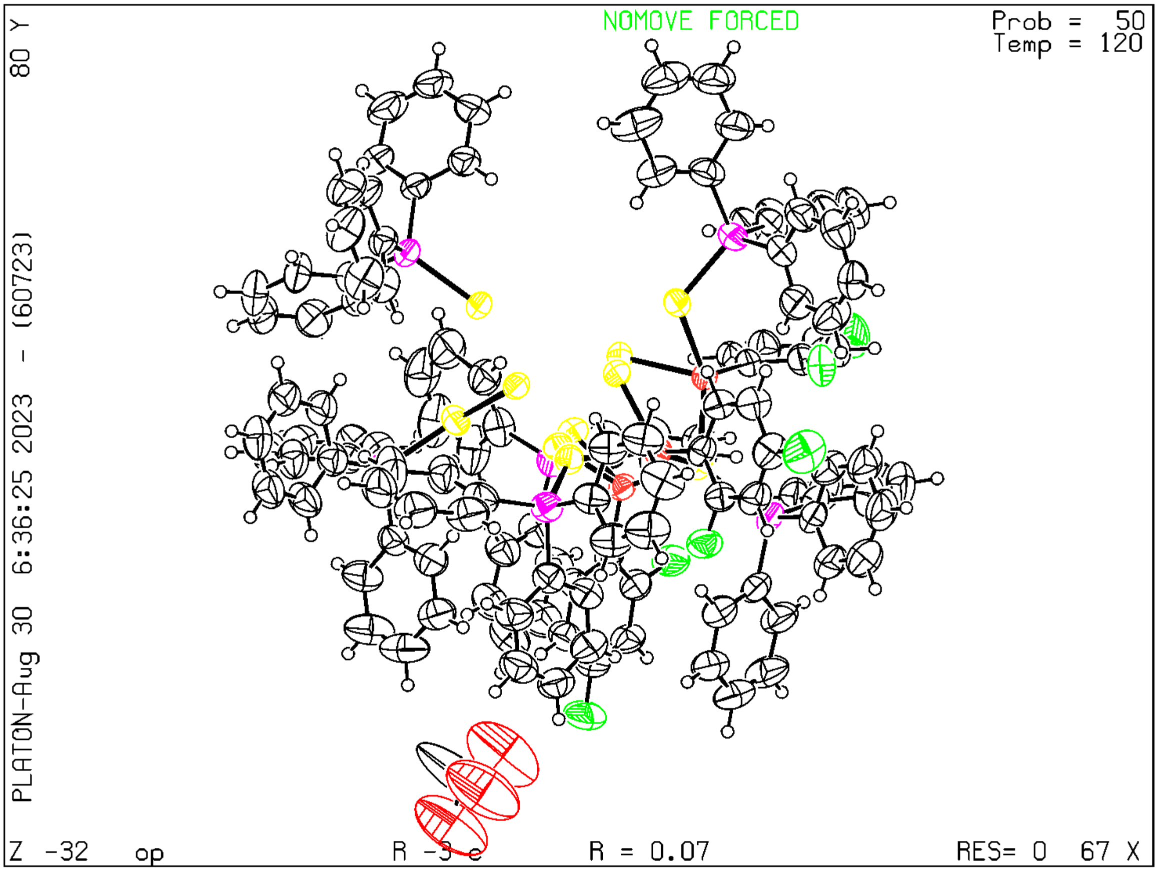

Supplement: Supplementary file 2 — Supporting Information [file ADVS-11-2307085-s002.zip › advs202307085-sup-0002-cif/checkcif-Cu14.pdf]
