# Supplementary material for: Total Structure, Structural Transformation and Catalytic Hydrogenation of [Cu41(SC6H3F2)15Cl3(P(PhF)3)6(H)25]2− Constructed from Twisted Cu13 Units
Source: Adv Sci (Weinh). 2023 Dec 8;11(7):2307085. doi: 10.1002/advs.202307085 (PMC10870033; doi:10.1002/advs.202307085)

checkCIF/PLATON report

You have not supplied any structure factors. As a result the full set of tests cannot be run.

THIS REPORT IS FOR GUIDANCE ONLY. IF USED AS PART OF A REVIEW PROCEDURE FOR PUBLICATION, IT SHOULD NOT REPLACE THE EXPERTISE OF AN EXPERIENCED CRYSTALLOGRAPHIC REFEREE.

No syntax errors found. CIF dictionary Interpreting this report

Datablock: cu41_sq

```
Bond precision:   C-C = 0.0148 A          Wavelength=1.54186

Cell:             a=20.3129(15)     b=33.065(2)        c=36.168(2)
                  alpha=90          beta=90.626(6)     gamma=90
Temperature:      120 K
```

	Calculated	Reported
Volume	24291(3)	24291(3)
Space group	P 21/c	P 21/c
Hall group	-P 2ybc	-P 2ybc
Moiety formula	C198 H117 Cu30 F48 P6 S15, 3(Cl Cu2), 3(C H2 Cl2), 5(Cu) [+ sol	C198 H117 Cu30 F48 P6 S15, 3(Cl Cu2), 3(C H2 Cl2), 5(Cu) [+ sol
Sum formula	C201 H123 Cl9 Cu41 F48 P6 S15 [+ solvent]	C201 H123 Cl9 Cu41 F48 P6 S15 [+ solvent]
Mr	7041.26	7040.90
Dx,g cm-3	1.925	1.925
Z	4	4
Mu (mm-1)	6.921	6.921
F000	13732.0	13732.0
F000'	13476.62	
h,k,lmax	23,38,42	23,38,42
Nref	41334	40053
Tmin,Tmax	0.273,0.288	0.310,0.370
Tmin'	0.175	

```
Correction method= # Reported T Limits: Tmin=0.310 Tmax=0.370
AbsCorr = MULTI-SCAN

Data completeness= 0.969          Theta(max)= 64.999
```

R(reflections)= 0.0636(21174) | wR2(reflections)= 0.1264(40053)

S = 1.034 | Npar= 2857

The following ALERTS were generated. Each ALERT has the format
test-name_ALERT_alert-type_alert-level.
Click on the hyperlinks for more details of the test.

Alert level A

PLAT307_ALERT_2_A Isolated Metal Atom found in Structure (Unusual) Cu10 Check
PLAT307_ALERT_2_A Isolated Metal Atom found in Structure (Unusual) Cu25 Check
PLAT307_ALERT_2_A Isolated Metal Atom found in Structure (Unusual) Cu35 Check
PLAT307_ALERT_2_A Isolated Metal Atom found in Structure (Unusual) Cu36 Check
PLAT307_ALERT_2_A Isolated Metal Atom found in Structure (Unusual) Cu40 Check
PLAT308_ALERT_2_A Single Bonded Metal Atom in Structure (Unusual) Cu2 Check
PLAT308_ALERT_2_A Single Bonded Metal Atom in Structure (Unusual) Cu3 Check
PLAT308_ALERT_2_A Single Bonded Metal Atom in Structure (Unusual) Cu6 Check
PLAT308_ALERT_2_A Single Bonded Metal Atom in Structure (Unusual) Cu7 Check
PLAT308_ALERT_2_A Single Bonded Metal Atom in Structure (Unusual) Cu8 Check
PLAT308_ALERT_2_A Single Bonded Metal Atom in Structure (Unusual) Cu11 Check
PLAT308_ALERT_2_A Single Bonded Metal Atom in Structure (Unusual) Cu24 Check
PLAT308_ALERT_2_A Single Bonded Metal Atom in Structure (Unusual) Cu28 Check
PLAT308_ALERT_2_A Single Bonded Metal Atom in Structure (Unusual) Cu30 Check
PLAT308_ALERT_2_A Single Bonded Metal Atom in Structure (Unusual) Cu33 Check
PLAT308_ALERT_2_A Single Bonded Metal Atom in Structure (Unusual) Cu38 Check
PLAT308_ALERT_2_A Single Bonded Metal Atom in Structure (Unusual) Cu41 Check
PLAT308_ALERT_2_A Single Bonded Metal Atom in Structure (Unusual) Cu5 Check
PLAT308_ALERT_2_A Single Bonded Metal Atom in Structure (Unusual) Cu29 Check
PLAT308_ALERT_2_A Single Bonded Metal Atom in Structure (Unusual) Cu9 Check
PLAT308_ALERT_2_A Single Bonded Metal Atom in Structure (Unusual) Cu18 Check
PLAT308_ALERT_2_A Single Bonded Metal Atom in Structure (Unusual) Cu1 Check
PLAT308_ALERT_2_A Single Bonded Metal Atom in Structure (Unusual) Cu22 Check

---

Alert level C

ABSTY02_ALERT_1_C An _exptl_absorpt_correction_type has been given without
a literature citation. This should be contained in the
_exptl_absorpt_process_details field.
Absorption correction given as multi-scan
THETM01_ALERT_3_C The value of sine(theta_max)/wavelength is less than 0.590
Calculated sin(theta_max)/wavelength = 0.5878
PLAT029_ALERT_3_C _diffrn_measured_fraction_theta_full value Low . 0.969 Why?
PLAT234_ALERT_4_C Large Hirshfeld Difference C13 --C14 . 0.17 Ang.
PLAT234_ALERT_4_C Large Hirshfeld Difference C29 --C30 . 0.16 Ang.
PLAT234_ALERT_4_C Large Hirshfeld Difference C34 --C35 . 0.18 Ang.
PLAT234_ALERT_4_C Large Hirshfeld Difference C41 --C42 . 0.16 Ang.
PLAT234_ALERT_4_C Large Hirshfeld Difference C43 --C44 . 0.18 Ang.
PLAT234_ALERT_4_C Large Hirshfeld Difference C43 --C48 . 0.17 Ang.
PLAT234_ALERT_4_C Large Hirshfeld Difference C47 --C48 . 0.16 Ang.
PLAT234_ALERT_4_C Large Hirshfeld Difference C49 --C54 . 0.16 Ang.
PLAT234_ALERT_4_C Large Hirshfeld Difference C51 --C52 . 0.22 Ang.
PLAT234_ALERT_4_C Large Hirshfeld Difference C53 --C54 . 0.16 Ang.
PLAT234_ALERT_4_C Large Hirshfeld Difference C55 --C56 . 0.17 Ang.
PLAT234_ALERT_4_C Large Hirshfeld Difference C56 --C57 . 0.16 Ang.

```
PLAT234_ALERT_4_C Large Hirshfeld Difference C62      --C63      .       0.20 Ang.
PLAT234_ALERT_4_C Large Hirshfeld Difference C67      --C68      .       0.16 Ang.
PLAT234_ALERT_4_C Large Hirshfeld Difference C68      --C69      .       0.19 Ang.
PLAT234_ALERT_4_C Large Hirshfeld Difference C69      --C70      .       0.17 Ang.
PLAT234_ALERT_4_C Large Hirshfeld Difference C70      --C71      .       0.18 Ang.
PLAT234_ALERT_4_C Large Hirshfeld Difference C88      --C89      .       0.16 Ang.
PLAT234_ALERT_4_C Large Hirshfeld Difference C91      --C96      .       0.17 Ang.
PLAT234_ALERT_4_C Large Hirshfeld Difference C97      --C102     .       0.16 Ang.
PLAT234_ALERT_4_C Large Hirshfeld Difference C103     --C108     .       0.19 Ang.
PLAT234_ALERT_4_C Large Hirshfeld Difference C105     --C106     .       0.17 Ang.
PLAT234_ALERT_4_C Large Hirshfeld Difference C109     --C110     .       0.18 Ang.
PLAT234_ALERT_4_C Large Hirshfeld Difference C112     --C113     .       0.16 Ang.
PLAT234_ALERT_4_C Large Hirshfeld Difference C116     --C117     .       0.17 Ang.
PLAT234_ALERT_4_C Large Hirshfeld Difference C117     --C118     .       0.16 Ang.
PLAT234_ALERT_4_C Large Hirshfeld Difference C129     --C130     .       0.19 Ang.
PLAT234_ALERT_4_C Large Hirshfeld Difference C135     --C136     .       0.19 Ang.
PLAT234_ALERT_4_C Large Hirshfeld Difference C137     --C138     .       0.18 Ang.
PLAT234_ALERT_4_C Large Hirshfeld Difference C145     --C146     .       0.18 Ang.
PLAT234_ALERT_4_C Large Hirshfeld Difference C145     --C150     .       0.23 Ang.
PLAT234_ALERT_4_C Large Hirshfeld Difference C148     --C149     .       0.22 Ang.
PLAT234_ALERT_4_C Large Hirshfeld Difference C149     --C150     .       0.21 Ang.
PLAT234_ALERT_4_C Large Hirshfeld Difference C157     --C158     .       0.17 Ang.
PLAT234_ALERT_4_C Large Hirshfeld Difference C157     --C162     .       0.16 Ang.
PLAT234_ALERT_4_C Large Hirshfeld Difference C163     --C168     .       0.20 Ang.
PLAT234_ALERT_4_C Large Hirshfeld Difference C165     --C166     .       0.16 Ang.
PLAT234_ALERT_4_C Large Hirshfeld Difference C171     --C172     .       0.23 Ang.
PLAT234_ALERT_4_C Large Hirshfeld Difference C176     --C177     .       0.16 Ang.
PLAT234_ALERT_4_C Large Hirshfeld Difference C177     --C178     .       0.19 Ang.
PLAT234_ALERT_4_C Large Hirshfeld Difference C182     --C183     .       0.18 Ang.
PLAT234_ALERT_4_C Large Hirshfeld Difference C184     --C185     .       0.21 Ang.
PLAT234_ALERT_4_C Large Hirshfeld Difference C187     --C188     .       0.16 Ang.
PLAT234_ALERT_4_C Large Hirshfeld Difference C193     --C194     .       0.16 Ang.
PLAT234_ALERT_4_C Large Hirshfeld Difference C193     --C198     .       0.16 Ang.
PLAT234_ALERT_4_C Large Hirshfeld Difference C195     --C196     .       0.18 Ang.
PLAT234_ALERT_4_C Large Hirshfeld Difference C197     --C198     .       0.20 Ang.
PLAT241_ALERT_2_C High   'MainMol' Ueq as Compared to Neighbors of        C41 Check
PLAT241_ALERT_2_C High   'MainMol' Ueq as Compared to Neighbors of        C44 Check
PLAT241_ALERT_2_C High   'MainMol' Ueq as Compared to Neighbors of        C59 Check
PLAT241_ALERT_2_C High   'MainMol' Ueq as Compared to Neighbors of       C147 Check
PLAT241_ALERT_2_C High   'MainMol' Ueq as Compared to Neighbors of       C173 Check
PLAT241_ALERT_2_C High   'MainMol' Ueq as Compared to Neighbors of       C191 Check
PLAT242_ALERT_2_C Low    'MainMol' Ueq as Compared to Neighbors of       C100 Check
PLAT242_ALERT_2_C Low    'MainMol' Ueq as Compared to Neighbors of       C150 Check
PLAT242_ALERT_2_C Low    'MainMol' Ueq as Compared to Neighbors of       C172 Check
PLAT244_ALERT_4_C Low    'Solvent' Ueq as Compared to Neighbors of       C200 Check
PLAT260_ALERT_2_C Large Average Ueq of Residue Including       Cl4      0.128 Check
PLAT260_ALERT_2_C Large Average Ueq of Residue Including       Cl6      0.115 Check
PLAT334_ALERT_2_C Small <C-C> Benzene Dist.   C61      -C66      .       1.36 Ang.
PLAT334_ALERT_2_C Small <C-C> Benzene Dist.   C115     -C120     .       1.37 Ang.
PLAT341_ALERT_3_C Low Bond Precision on  C-C Bonds ...............    0.01481 Ang.
```

Alert level G

```
FORMU01_ALERT_1_G  There is a discrepancy between the atom counts in the
            _chemical_formula_sum and _chemical_formula_moiety. This is
            usually due to the moiety formula being in the wrong format.
            Atom count from _chemical_formula_sum:   C201 H123 Cl9 Cu41 F48 P6 S15
```

```
            Atom count from _chemical_formula_moiety:C201 H123 Cl9 Cu36 F48 P6 S15
CELLZ01_ALERT_1_G Difference between formula and atom_site contents detected.
CELLZ01_ALERT_1_G ALERT: Large difference may be due to a
            symmetry error - see SYMMG tests
           From the CIF: _cell_formula_units_Z    4
           From the CIF: _chemical_formula_sum  C201 H123 Cl9 Cu41 F48 P6 S15 [+ s
           TEST: Compare cell contents of formula and atom_site data

           atom    Z*formula  cif sites diff
           C        804.00    804.00    0.00
           H        492.00    492.00    0.00
           Cl        36.00     36.00    0.00
           Cu       164.00    164.00    0.00
           F        192.00    192.00    0.00
           P         24.00     24.00    0.00
           S         60.00     60.00    0.00
           [+         4.00      0.00    4.00
           solve      4.00      0.00    4.00
PLAT003_ALERT_2_G Number of Uiso or Uij Restrained non-H Atoms ...         29 Report
PLAT177_ALERT_4_G The CIF-Embedded .res File Contains DELU Records          3 Report
PLAT192_ALERT_3_G A Non-default DELU Restraint Value for First Par     0.0010 Report
PLAT192_ALERT_3_G A Non-default DELU Restraint Value for First Par     0.0010 Report
PLAT192_ALERT_3_G A Non-default DELU Restraint Value for First Par     0.0010 Report
PLAT232_ALERT_2_G Hirshfeld Test Diff (M-X)  Cu4      --S3       .        7.5 s.u.
PLAT232_ALERT_2_G Hirshfeld Test Diff (M-X)  Cu7      --S4       .        5.4 s.u.
PLAT232_ALERT_2_G Hirshfeld Test Diff (M-X)  Cu15     --S15      .        6.1 s.u.
PLAT232_ALERT_2_G Hirshfeld Test Diff (M-X)  Cu16     --S6       .       12.2 s.u.
PLAT232_ALERT_2_G Hirshfeld Test Diff (M-X)  Cu16     --P2       .       11.9 s.u.
PLAT232_ALERT_2_G Hirshfeld Test Diff (M-X)  Cu20     --S13      .        5.6 s.u.
PLAT232_ALERT_2_G Hirshfeld Test Diff (M-X)  Cu20     --S14      .        5.2 s.u.
PLAT232_ALERT_2_G Hirshfeld Test Diff (M-X)  Cu21     --S4       .        6.6 s.u.
PLAT232_ALERT_2_G Hirshfeld Test Diff (M-X)  Cu21     --S5       .        6.8 s.u.
PLAT232_ALERT_2_G Hirshfeld Test Diff (M-X)  Cu26     --S6       .        5.3 s.u.
PLAT232_ALERT_2_G Hirshfeld Test Diff (M-X)  Cu27     --S10      .        8.9 s.u.
PLAT232_ALERT_2_G Hirshfeld Test Diff (M-X)  Cu31     --S4       .        5.6 s.u.
PLAT232_ALERT_2_G Hirshfeld Test Diff (M-X)  Cu32     --S12      .        9.5 s.u.
PLAT232_ALERT_2_G Hirshfeld Test Diff (M-X)  Cu33     --S15      .        5.6 s.u.
PLAT232_ALERT_2_G Hirshfeld Test Diff (M-X)  Cu34     --S10      .        5.5 s.u.
PLAT232_ALERT_2_G Hirshfeld Test Diff (M-X)  Cu37     --S3       .        7.2 s.u.
PLAT232_ALERT_2_G Hirshfeld Test Diff (M-X)  Cu37     --S8       .        8.8 s.u.
PLAT232_ALERT_2_G Hirshfeld Test Diff (M-X)  Cu39     --S7       .        5.4 s.u.
PLAT232_ALERT_2_G Hirshfeld Test Diff (M-X)  Cu39     --P3       .        6.6 s.u.
PLAT335_ALERT_2_G Check Large C6 Ring C-C Range C19      -C24             0.20 Ang.
PLAT335_ALERT_2_G Check Large C6 Ring C-C Range C49      -C54             0.17 Ang.
PLAT434_ALERT_2_G Short Inter HL..HL Contact Cl6      ..F3       .       3.10 Ang.
                                              1+x,y,z  =      1_655 Check
PLAT434_ALERT_2_G Short Inter HL..HL Contact F1       ..F5       .       2.77 Ang.
                                             -1+x,y,z  =      1_455 Check
PLAT606_ALERT_4_G Solvent Accessible VOID(S) in Structure ........          ! Info
PLAT764_ALERT_4_G Overcomplete CIF Bond List Detected (Rep/Expd) .       1.37 Ratio
PLAT794_ALERT_5_G Tentative Bond Valency for Cu1       (I)       .       0.22 Info
PLAT794_ALERT_5_G Tentative Bond Valency for Cu2       (I)       .       0.23 Info
PLAT794_ALERT_5_G Tentative Bond Valency for Cu3       (I)       .       0.24 Info
PLAT794_ALERT_5_G Tentative Bond Valency for Cu4       (I)       .       0.62 Info
PLAT794_ALERT_5_G Tentative Bond Valency for Cu5       (I)       .       0.23 Info
PLAT794_ALERT_5_G Tentative Bond Valency for Cu6       (I)       .       0.23 Info
PLAT794_ALERT_5_G Tentative Bond Valency for Cu7       (I)       .       0.24 Info
```

```
PLAT794_ALERT_5_G Tentative Bond Valency for Cu8       (I)       .       0.23 Info
PLAT794_ALERT_5_G Tentative Bond Valency for Cu9       (I)       .       0.32 Info
PLAT794_ALERT_5_G Tentative Bond Valency for Cu11      (I)       .       0.26 Info
PLAT794_ALERT_5_G Tentative Bond Valency for Cu12      (I)       .       0.73 Info
PLAT794_ALERT_5_G Tentative Bond Valency for Cu13      (I)       .       0.72 Info
PLAT794_ALERT_5_G Tentative Bond Valency for Cu14      (I)       .       0.72 Info
PLAT794_ALERT_5_G Tentative Bond Valency for Cu15      (I)       .       0.73 Info
PLAT794_ALERT_5_G Tentative Bond Valency for Cu16      (I)       .       0.94 Info
PLAT794_ALERT_5_G Tentative Bond Valency for Cu17      (I)       .       0.62 Info
PLAT794_ALERT_5_G Tentative Bond Valency for Cu18      (I)       .       0.23 Info
PLAT794_ALERT_5_G Tentative Bond Valency for Cu19      (I)       .       0.62 Info
PLAT794_ALERT_5_G Tentative Bond Valency for Cu20      (I)       .       0.93 Info
PLAT794_ALERT_5_G Tentative Bond Valency for Cu21      (I)       .       0.71 Info
PLAT794_ALERT_5_G Tentative Bond Valency for Cu22      (I)       .       0.33 Info
PLAT794_ALERT_5_G Tentative Bond Valency for Cu23      (I)       .       0.61 Info
PLAT794_ALERT_5_G Tentative Bond Valency for Cu24      (I)       .       0.26 Info
PLAT794_ALERT_5_G Tentative Bond Valency for Cu26      (I)       .       0.71 Info
PLAT794_ALERT_5_G Tentative Bond Valency for Cu27      (I)       .       0.98 Info
PLAT794_ALERT_5_G Tentative Bond Valency for Cu28      (I)       .       0.27 Info
PLAT794_ALERT_5_G Tentative Bond Valency for Cu29      (I)       .       0.31 Info
PLAT794_ALERT_5_G Tentative Bond Valency for Cu30      (I)       .       0.23 Info
PLAT794_ALERT_5_G Tentative Bond Valency for Cu31      (I)       .       0.97 Info
PLAT794_ALERT_5_G Tentative Bond Valency for Cu32      (I)       .       0.96 Info
PLAT794_ALERT_5_G Tentative Bond Valency for Cu33      (I)       .       0.27 Info
PLAT794_ALERT_5_G Tentative Bond Valency for Cu34      (I)       .       0.62 Info
PLAT794_ALERT_5_G Tentative Bond Valency for Cu37      (I)       .       0.59 Info
PLAT794_ALERT_5_G Tentative Bond Valency for Cu38      (I)       .       0.26 Info
PLAT794_ALERT_5_G Tentative Bond Valency for Cu39      (I)       .       0.94 Info
PLAT794_ALERT_5_G Tentative Bond Valency for Cu41      (I)       .       0.28 Info
PLAT860_ALERT_3_G Number of Least-Squares Restraints ............         18 Note
PLAT869_ALERT_4_G ALERTS Related to the Use of SQUEEZE  Suppressed         ! Info
PLAT883_ALERT_1_G No Info/Value for _atom_sites_solution_primary .     Please Do !
PLAT933_ALERT_2_G Number of HKL-OMIT Records in Embedded .res File         66 Note
PLAT941_ALERT_3_G Average HKL Measurement Multiplicity ..........        3.0 Low
PLAT965_ALERT_2_G The SHELXL WEIGHT Optimisation has not Converged     Please Check
PLAT967_ALERT_5_G Note: Two-Theta Cutoff Value in Embedded .res ..      130.0 Degree
```

---

 23 **ALERT level A** = Most likely a serious problem - resolve or explain
 0 **ALERT level B** = A potentially serious problem, consider carefully
 65 **ALERT level C** = Check. Ensure it is not caused by an omission or oversight
 76 **ALERT level G** = General information/check it is not something unexpected

 5 ALERT type 1 CIF construction/syntax error, inconsistent or missing data
 62 ALERT type 2 Indicator that the structure model may be wrong or deficient
 8 ALERT type 3 Indicator that the structure quality may be low
 52 ALERT type 4 Improvement, methodology, query or suggestion
 37 ALERT type 5 Informative message, check

It is advisable to attempt to resolve as many as possible of the alerts in all categories. Often the minor alerts point to easily fixed oversights, errors and omissions in your CIF or refinement strategy, so attention to these fine details can be worthwhile. In order to resolve some of the more serious problems it may be necessary to carry out additional measurements or structure refinements. However, the purpose of your study may justify the reported deviations and the more serious of these should normally be commented upon in the discussion or experimental section of a paper or in the "special_details" fields of the CIF. checkCIF was carefully designed to identify outliers and unusual parameters, but every test has its limitations and alerts that are not important in a particular case may appear. Conversely, the absence of alerts does not guarantee there are no aspects of the results needing attention. It is up to the individual to critically assess their own results and, if necessary, seek expert advice.

Publication of your CIF in IUCr journals

A basic structural check has been run on your CIF. These basic checks will be run on all CIFs submitted for publication in IUCr journals (*Acta Crystallographica*, *Journal of Applied Crystallography*, *Journal of Synchrotron Radiation*); however, if you intend to submit to *Acta Crystallographica Section C* or *E* or *IUCrData*, you should make sure that full publication checks are run on the final version of your CIF prior to submission.

Publication of your CIF in other journals

Please refer to the *Notes for Authors* of the relevant journal for any special instructions relating to CIF submission.

PLATON version of 06/07/2023; check.def file version of 30/06/2023

Datablock cu41_sq - ellipsoid plot

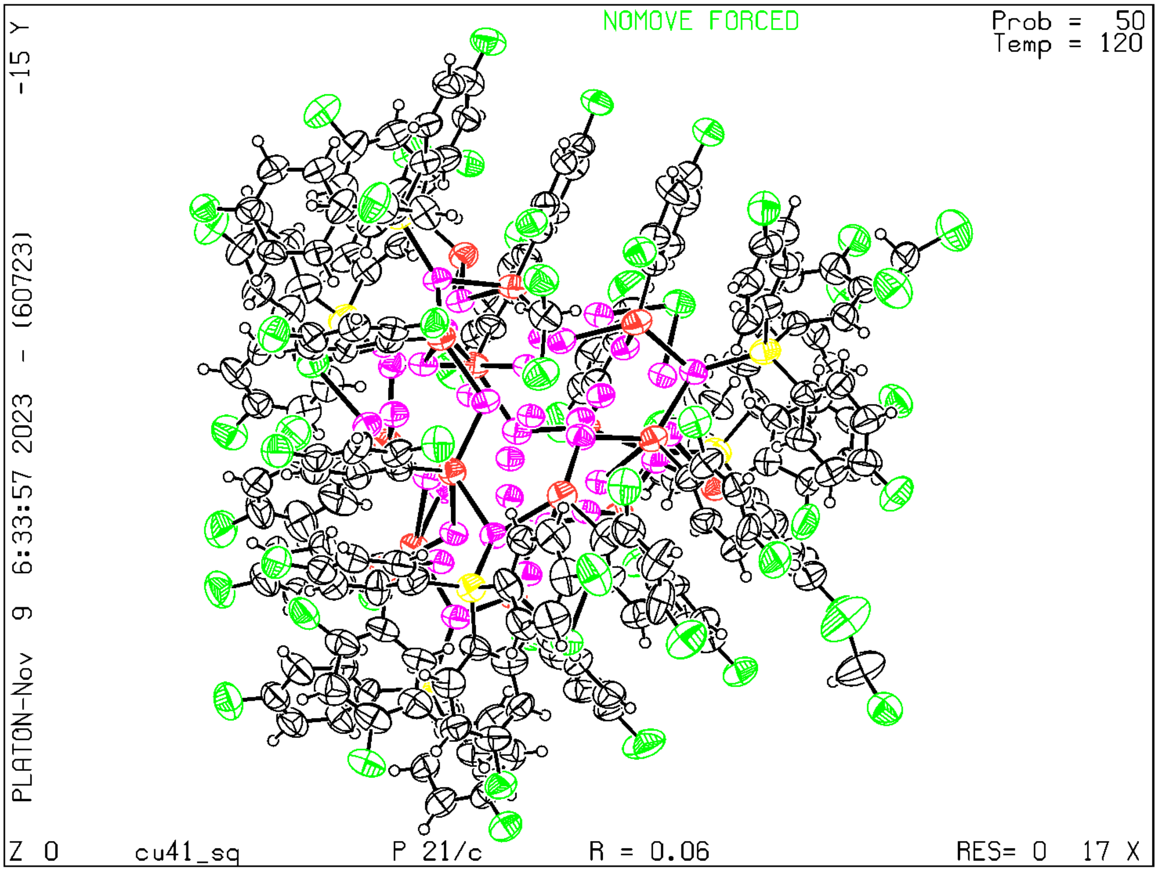

Supplement: Supplementary file 2 — Supporting Information [file ADVS-11-2307085-s002.zip › advs202307085-sup-0002-cif/checkcif-Cu41-revised-1109.pdf]
